# Chronic Obstructive Pulmonary Disease Mortality and Hospitalization during the COVID-19 Pandemic Compared with before the Pandemic: A Systematic Review and Meta-Analysis

**DOI:** 10.3390/jpm14030296

**Published:** 2024-03-10

**Authors:** Chiwon Ahn, Yeonkyung Park

**Affiliations:** 1 Department of Emergency Medicine, College of Medicine, Chung-Ang University, Seoul 06974, Republic of Korea; cahn@cau.ac.kr; 2 Division of Pulmonary and Critical Care Medicine, Department of Internal Medicine, Veterans Health Service Medical Center, Seoul 05368, Republic of Korea; 3 Department of Internal Medicine, College of Medicine, Hanyang University, Seoul 04763, Republic of Korea

**Keywords:** chronic obstructive pulmonary disease, COVID-19, hospitalization, mortality

## Abstract

This study aimed to assess the impact of the pandemic on hospitalization and mortality rates among patients with acute exacerbation of chronic obstructive pulmonary disease (AECOPD). We conducted a systematic search across three medical databases for studies comparing the AECOPD mortality and hospitalization rates during the COVID-19 pandemic with those before the pandemic, up until December 2023. Using the Preferred Reporting Items for Systematic Reviews and Meta-Analyses and Meta-analysis of Observational Studies in Epidemiology guidelines, we performed a meta-analysis with a random-effects model to pool odds ratios (ORs), 95% confidence intervals (CIs), and heterogeneity (I^2^). From 4689 records, 21 studies met our inclusion criteria. Our analysis revealed a significant increase in in-hospital mortality during the pandemic (pooled OR = 1.27, 95% CI = 1.17–1.39, I^2^ = 50%). Subgroup analysis highlighted a more pronounced mortality risk in single-center studies and smaller populations. Conversely, hospitalization rates for AECOPD significantly declined during the pandemic (pooled OR = 0.39, 95% CI = 0.18–0.85, I^2^ = 99%). The study demonstrates that during the COVID-19 pandemic, there was a substantial decrease in hospital admissions for AECOPD and an increase in in-hospital deaths. This shows that better healthcare plans and pandemic preparedness are needed to help people with chronic conditions.

## 1. Introduction

The COVID-19 pandemic has severely strained healthcare systems, resulting in resource diversion and potential collapse [1,2,3,4]. The crisis triggered a care deficit for non-COVID diseases such as acute myocardial infarction, stroke, out-of-hospital cardiac arrest, and sepsis, with delayed diagnoses and treatments due to enhanced hospital screening procedures. Additionally, there was a marked decrease in hospitalizations for non-COVID-19 diseases [4,5,6,7,8]. This was partially caused by hospitals reallocating resources to handle the spike in COVID-19 cases, and partially because patients delayed or refused treatment for fear of contracting the virus in the healthcare facility [9,10]. Significant issues with patients with chronic conditions could occur because of decreased hospitalizations for non-COVID-19 diseases, possibly leading to disease progression and worsening medical outcomes.

Chronic obstructive pulmonary disease (COPD) is a leading cause of mortality, morbidity, and healthcare costs [11,12]. Hospitalizations for an acute exacerbation of COPD (AECOPD) are associated with accelerated lung function decline, worsened quality of life, increased future exacerbation risk, and higher mortality rates [13]. Patients with frequent exacerbations are at significantly elevated risk [14]. Moreover, severe AECOPD is associated with a poor quality of life, increased mortality, and cardiovascular complications. In the context of COVID-19, patients with COPD face a heightened risk of severe illness, delayed medical care, disrupted management, and increased anxiety. Pappe et al. demonstrated that the COVID-19 pandemic was associated with decreased hospitalization and a modest increase in mortality among hospitalized patients with AECOPD [15]. However, no previous meta-analysis has compared the mortality rates among patients with COPD between the pandemic and pre-pandemic eras.

We conducted a comprehensive meta-analysis to elucidate the indirect consequences of the COVID-19 pandemic on the care and outcomes of AECOPD, contrasting mortality and hospitalization rates during the pandemic with those from the before the pandemic. This analysis is crucial to comprehend the consequences that disrupted healthcare services have on chronic conditions such as COPD. Our objective is to provide insights into these patterns to enhance clinical practices and clarify healthcare policies that will assist in managing the difficulties presented by pandemics and ensuring the uninterrupted provision of care for chronic illnesses.

## 2. Materials and Methods

### 2.1. Reporting Guidelines and Protocol Registration

This study was based on the Preferred Reporting Items for Systematic Reviews and Meta-Analyses and the Meta-analysis of Observational Studies in Epidemiology guidelines for reporting information from observational studies [16,17]. The review protocol was prospectively registered in PROSPERO at http://www.crd.york.ac.uk/PROSPERO/; registration number: CRD42022372883 (accessed on 10 February 2024).

### 2.2. Search Strategy

We systematically searched three electronic databases (MEDLINE, Embase, and Cochrane Library) for relevant studies on hospitalized patients with AECOPD during the COVID-19 pandemic compared with those before the pandemic through December 2023. Medical subject headings (MeSH), Embase subject headings, and text words were included in the search strategy. We combined the MeSH terms and free terms related to “COPD” and “COVID-19”. A detailed search strategy is presented in Supplementary Table S1.

### 2.3. Study Selection and Data Extraction

We screened the titles and abstracts, identified the article types, and excluded irrelevant studies. First, duplicates were excluded. If the title, author, and publication year of a paper were the same, we judged it as a duplicate. We excluded articles with the following criteria: reviews, case reports, case series, editorials, comments, meta-analyses, irrelevant populations, and inappropriate comparisons. Studies involving patients younger than 18 years were excluded. If two reviewers disagreed regarding the study selection, differences were discussed until a consensus was reached. Finally, we included studies that assessed hospitalization for AECOPD during the COVID-19 pandemic and compared them with those before the pandemic. We subsequently reviewed the full texts of potentially relevant articles that met the inclusion criteria.

Two reviewers independently extracted the following information from the included studies: authors, year of publication, region of study, study period (during and before the pandemic), population size, age, sex, hospitalization rate, and mortality. Discrepancies between the reviewers were resolved by consensus.

The primary outcome of the study was mortality, which included mortality that occurred both within the hospital and at different time intervals after discharge. Additionally, details about hospitalization rates were collected.

### 2.4. Quality Assessment in Individual Studies

The Newcastle–Ottawa Scale is a quality assessment tool used to evaluate nonrandomized studies based on an eight-item score divided into three domains [18]. Each article was evaluated using a score based on the number of stars from three perspectives: (a) selection (maximum: four stars), (b) comparability (maximum: two stars), and (c) outcome (maximum: three stars).

### 2.5. Statistical Analysis

This meta-analysis investigated the hospitalization and mortality of patients with COPD exacerbations during the COVID-19 pandemic compared to those before the pandemic. For dichotomous variables, we calculated the pooled OR with a 95% confidence interval (CI) using a random-effects model. We estimated the proportion of between-study inconsistencies using the I^2^ statistic to assess the heterogeneity. We considered I^2^ values of 25%, 50%, and 75% as low, moderate, and high heterogeneity, respectively [19]. We conducted planned subgroup analyses based on the number of hospitals conducting a study (multicenter or single center), article type (original article or letter/conference abstract), and population size (≥1000 population or <1000 population). In addition, the subgroup analysis of hospitalization included the same cohort of two groups (same patients or separate patients). We performed a meta-analysis and quality assessment of the included studies using Review Manager version 5.4 (Cochrane Collaboration 2012, Nordic Cochrane Centre, Copenhagen, Denmark), and R (version 4.3.1, The R Foundation for Statistical Computing, Vienna, Austria) software packages “meta” (version 4.11-0) and “metaphor” (version 2.1-0), respectively, considering a *p*-value of < 0.05 to be statistically significant. Funnel plots were used to assess publication bias.

## 3. Results

### 3.1. Baseline Characteristics

**Figure 1:**
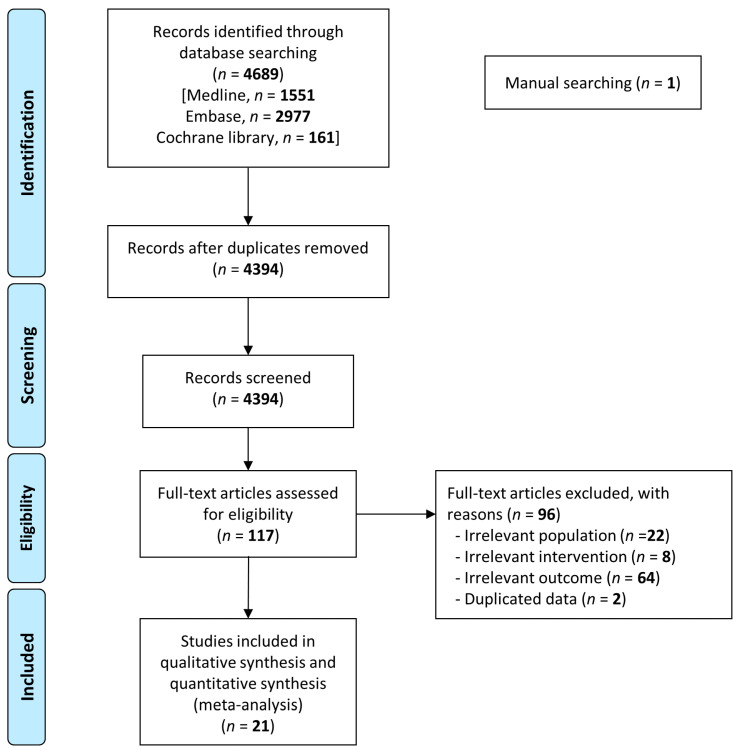
Preferred Reporting Items for Systematic Reviews and Meta-analyses flow chart of included and excluded studies.

In total, we identified 4689 articles, of which 4394 articles remained after the duplicates were removed. After assessing their titles and abstracts, we excluded 4277 articles for irrelevance and retrieved the full texts of the 117 remaining relevant studies. We excluded studies with an irrelevant population (*n* = 22), irrelevant intervention (*n* = 8), irrelevant outcome (*n* = 64), or duplicated data (*n* = 2). Finally, we conducted a meta-analysis and systematic review of 21 eligible studies (Figure 1). 

The studies included eleven with the outcome of mortality [20,21,22,23,24,25,26,27,28,29,30] and eleven with the outcome of hospitalization for AECOPD [22,31,32,33,34,35,36,37,38,39,40]. Dang et al. reported the outcomes of mortality and hospitalization rates for AECOPD [22]. Twelve studies had a European research region, including France, Germany, Hungary, Malta, Portugal, Slovenia, Spain, Türkiye, and the United Kingdom [23,24,25,28,29,30,32,33,34,35,36,37]. There were eleven multicenter [22,23,25,26,28,29,30,32,35,39,40] and ten single-center studies. Fifteen studies were observational cohort studies [21,22,23,24,25,26,28,29,32,34,35,37,38,39], and six studies were letters or conference abstracts [20,27,30,34,37,40] (Table 1).

### 3.2. Quality Assessment

For the Newcastle–Ottawa Scale result, all studies had four points in the selection domain and one point in the outcome assessment. Eight studies had two additional points after adjusting for confounding factors [22,23,24,25,29,32,35,38] (Supplementary Table S2).

### 3.3. Mortality of AECOPD

**Figure 2:**
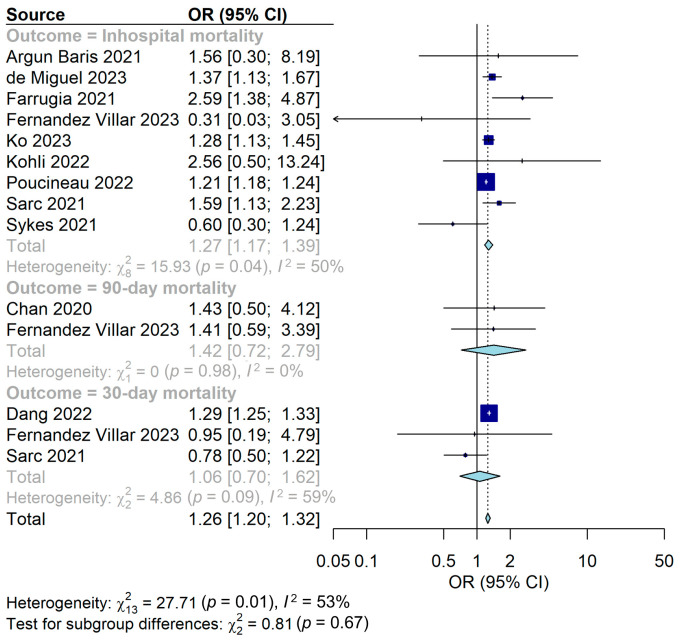
Forest plot for mortality of AECOPD during the COVID-19 pandemic compared with before the pandemic [20,21,22,23,24,25,26,27,28,29,30].

Among the eleven studies that reported mortality due to AECOPD, the nine studies showed the in-hospital mortality, three studies showed the 30-day mortality, and two studies showed the 90-day mortality. The in-hospital mortality was significantly high during the COVID-19 pandemic compared to before the pandemic (pooled OR = 1.27, 95% CI = 1.17–1.39, I^2^ = 50%) (Figure 2). However, the 30-day mortality and 90-day mortality were not significantly different (pooled OR = 1.06, 95% CI = 0.70–1.62, I^2^ = 59%; pooled OR = 1.42, 95% CI = 0.72–2.79, I^2^ = 0%) (Figure 2).

Because only studies with in-hospital mortality had sufficient numbers, subgroup analysis was performed. In the subgroup analysis according to the number of hospitals conducting a study, the OR for multicenter studies was 1.24 (95% CI = 1.17–1.31, I^2^ = 48%), and the OR for single-center studies was 2.45 (95% CI = 1.40–4.26, I^2^ = 0%) (Table 2 and Supplementary Figure S1A). In the subgroup analysis according to the sample size, the OR for large populations was 1.24 (95% CI = 1.17–1.32, I^2^ = 51%), and the OR for small populations was 2.18 (95% CI = 1.27–3.74, I^2^ = 8%) (Table 2 and Supplementary Figure S1B). In the subgroup analysis according to article type, the OR for original articles was 1.29 (95% CI = 1.18–1.42, I^2^ = 56%), and the OR for single-center studies was 1.05 (95% CI = 0.41–2.70, I^2^ = 36%) (Table 2 and Supplementary Figure S1C).

### 3.4. Hospitalization of AECOPD

**Figure 3:**
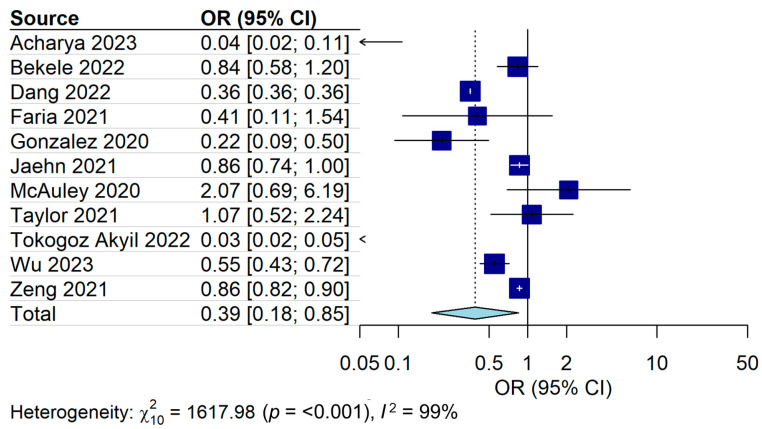
Forest plot for admission of AECOPD during the COVID-19 pandemic compared with before the pandemic [22,31,32,33,34,35,36,37,38,39,40].

Among the eleven studies showing the AECOPD hospitalization rate, it was significantly low during the COVID-19 pandemic compared to before the pandemic (pooled OR = 0.39, 95% CI = 0.18–0.85, I^2^ = 99%) (Figure 3). 

In the subgroup analysis according to the number of hospitals conducting a study, the OR for multicenter studies was 0.65 (95% CI = 0.46–0.93, I^2^ = 100%), and the OR for single-center studies was 0.24 (95% CI = 0.06–0.97, I^2^ = 95%) (Table 2 and Supplementary Figure S2A). In the subgroup analysis according to the sample size, the OR for large populations was 0.68 (95% CI = 0.44–1.05, I^2^ = 100%), and the OR for small populations was 0.27 (95% CI = 0.08–0.90, I^2^ = 96%) (Table 2 and Supplementary Figure S2B). In the subgroup analysis according to article type, the OR for original articles was 0.33 (95% CI = 0.12–0.92, I^2^ = 98%), and the OR for single-center studies was 0.61 (95% CI = 0.25–1.53, I^2^ = 81%) (Table 2 and Supplementary Figure S2C). In the subgroup analysis according to article type, the OR for original articles was 0.20 (95% CI = 0.04–0.90, I^2^ = 97%), and the OR for single-center studies was 0.70 (95% CI = 0.48–1.01, I^2^ = 100%) (Table 2 and Supplementary Figure S2D).

### 3.5. Publication Bias

We generated funnel plots for each outcome (refer to Supplementary Figure S3). Although the plots did not exhibit perfect symmetry, this does not necessarily indicate publication bias. To statistically assess the presence of publication bias, we performed Egger’s test. For the outcome “mortality”, the test yielded a t-value of 0.96, 9 degrees of freedom (df), and a *p*-value of 0.3626. As the *p*-value exceeds the conventional threshold of 0.05, the observed bias is not statistically significant, suggesting no evidence of publication bias in this part of the meta-analysis. Similarly, for the outcome “hospitalization”, the test resulted in a t-value of 0.36, 12 degrees of freedom (df), and a *p*-value of 0.7219. Given that the *p*-value is greater than the conventional threshold of 0.05, the observed bias is not statistically significant, indicating no evidence of publication bias in this part of the meta-analysis.

## 4. Discussion

This study aimed to evaluate the impact of the COVID-19 pandemic on the care and outcomes of individuals with COPD, with a focus on the indirect effects of the pandemic. COPD is marked by recurrent AECOPD, which often necessitates hospitalization and is linked to high mortality rates. The COVID-19 pandemic could have interrupted the standard management of AECOPD, potentially leading to delays in diagnosis, treatment, and follow-up [41]. To explore the effects of these disruptions, we performed a meta-analysis to compare the mortality and hospitalization rates for AECOPD during the pandemic to those before it. The findings indicate significant changes in both areas: there was a substantial decrease in the hospital admissions for AECOPD during the pandemic, which suggests that patients with COPD might have avoided seeking medical attention due to fears of COVID-19 infection. Furthermore, our analysis revealed an increase in in-hospital mortality rates among patients hospitalized for AECOPD during the pandemic, suggesting that either the severity of the disease had increased or the quality of care may have been compromised. Interestingly, the 30-day and 90-day mortality rates did not show significant differences, highlighting a complex interplay between immediate outcomes and longer-term survival.

The COVID-19 pandemic has significantly altered global healthcare systems, prompting a shift in resources and creating gaps in the treatment of non-COVID disorders, including COPD. Previous reports highlighted a marked decrease in hospital admissions for non-COVID conditions, along with reduced visits to emergency departments and primary care consultations for COPD [42,43,44]. Our meta-analysis, encompassing eleven studies focused on hospitalization rates, uncovered a significant reduction in hospital admissions for severe AECOPD during the COVID-19 era compared to the pre-pandemic period, indicating that fears of viral transmission, reallocation of healthcare resources, and potential decreases in respiratory viral infections and air pollution did not prevent a significant decline in hospitalizations for severe AECOPD. [9,10,43,45,46]. Severe AECOPD requiring hospital admission did not show a statistically significant decrease. This significant decrease suggests a complex interplay of factors, including possibly heightened patient reluctance to seek hospital care due to the risk of COVID-19 infection and the impact of pandemic-related changes in healthcare delivery on the management of AECOPD. Nonetheless, the results across the included studies showed a high degree of heterogeneity, reflecting variations in the study design, data sources, time frames, geographic locations, and definitions of AECOPD. These inconsistencies underscore the need for further investigation to better understand the nuanced effects of the COVID-19 pandemic on the management and outcomes of AECOPD.

Additionally, we observed increased mortality among patients with AECOPD during the pandemic (pooled OR = 1.25, 95% CI = 1.19–1.31), and subgroup analysis suggests that this increased in-hospital mortality risk was more pronounced in single-center studies and among smaller study populations. This implies that the quality and effectiveness of the therapy provided to patients who received treatment may have been compromised. Hospitalized patients typically have more severe cases, and the redirection of resources away from non-COVID disorders may have resulted in inadequate management of these patients, resulting in greater mortality rates [47]. Additionally, there is a lack of medical resources for distinguishing patients with COVID-19 infection from non-infectious patients [48]. Hospitalized patients with COVID-19 infection occupied intensive care units [49]. High-severity COVID-19 is more likely to require intensive care, a resource limited to hospitals caring for high levels of patients with COVID-19. Furthermore, delays in seeking treatment due to COVID-19 anxiety may have accelerated disease progression, leading to increased mortality [47]. Patients feared contracting COVID-19 infection during hospital visits during the pandemic, receiving hospital access later, and passing the critical time to be cured [50]. For these reasons, hospitalized patients tend to have more severe diseases and a higher risk of death.

These findings highlight the importance of maintaining the continuity of care for chronic diseases during pandemics. Efforts should be made to mitigate the effects of resource reallocation on non-COVID diseases, and alternative care delivery strategies, such as telemedicine, should be explored to ensure timely and effective care. Telemedicine can provide remote diagnosis, monitoring, and management of chronic diseases, reducing the need for face-to-face visits and minimizing the risk of exposure to COVID-19 [51,52]. Additionally, addressing concerns from patients about accessing healthcare during a pandemic is crucial, and effective risk communication strategies and infection control measures should be implemented in healthcare settings. Risk communication can inform and educate patients about the benefits and risks of seeking care, the availability and accessibility of services, and the safety protocols in place to prevent COVID-19 transmission [53]. Infection control measures can include screening, triage, isolation, personal protective equipment, ventilation, and disinfection [51,54]. These actions can help reduce the fear and anxiety of patients, increase their trust and confidence in the healthcare system, and improve their adherence to chronic disease management.

One of the challenges for patients with COPD during the COVID-19 pandemic is to maintain their self-management skills and behaviors, such as medication adherence, smoking cessation, physical activity, and symptom monitoring. Self-management is crucial for preventing and managing AECOPD, improving health outcomes, and reducing healthcare utilization [50,55]. However, the pandemic may have disrupted the usual delivery of and access to self-management programs for patients with COPD, such as pulmonary rehabilitation, education, and support groups. Therefore, remote delivery options, such as telehealth, online platforms, and mobile applications, have emerged as potential alternatives to provide self-management interventions for patients with COPD during the pandemic [56]. These options can offer convenience, flexibility, and safety for patients and healthcare providers, as well as reduce the barriers of distance, transportation, and availability of resources. However, there are also challenges and limitations of remote delivery options, such as technical issues, lack of personal interaction, privacy concerns, and digital literacy. Therefore, more research is needed to evaluate the effectiveness, feasibility, acceptability, and cost-effectiveness of remote delivery options for self-management programs for patients with COPD during and after the COVID-19 pandemic.

This article has several limitations that should be acknowledged. First, this article is based on a meta-analysis of observational studies, which may have inherent biases and confounding factors that affect the results. Therefore, the causal relationship between COPD and COVID-19 outcomes cannot be established. Second, in reflecting on the limitations of our study, we recognize that the majority of data were collected during the initial phase of the pandemic, which may limit the applicability of our findings to later stages where treatment strategies and public health responses may have evolved. Third, the severity of hospitalized COPD cases was not uniformly accounted for across the included studies, which may serve as a potential explanation for the observed increase in mortality rates. This oversight represents a critical gap in our analysis, and future studies should aim to incorporate severity indicators to better understand the factors contributing to mortality among hospitalized AECOPD patients during the pandemic. Fourth, this article did not include studies that reported adjusted risks for COPD and COVID-19 outcomes, which may have accounted for other potential risk factors, such as age, comorbidities, smoking status, and severity of COPD. Therefore, the true risks of COPD for COVID-19 outcomes may be overestimated or underestimated. Fifth, this article did not consider the heterogeneity of COPD and COVID-19 definitions, diagnosis, and management across different studies, settings, and countries. Additionally, we recognize that heterogeneity among the included studies, due to variations in treatment strategies for AECOPD, the severity of exacerbation of COPD cases, and differing COVID-19 status and responses across countries, represents a significant confounding factor potentially affecting the interpretation of our results. While our meta-analysis provides valuable insights into the indirect consequences of the COVID-19 pandemic on AECOPD care and outcomes, it is important to note that these findings should be interpreted with caution due to these limitations. Data heterogeneity stems from differences in healthcare policies, access to healthcare services, and the adoption of preventive measures during the pandemic across different countries. This variability underscores the need for country-specific analyses to better understand the impact of the pandemic on AECOPD management and outcomes. Finally, the inclusion of studies from diverse healthcare settings and treatment approaches (e.g., non-invasive ventilation, systemic corticosteroids, and antibiotics) reflects the real-world complexity of AECOPD management during the pandemic but also contributes to the observed heterogeneity. Future research should aim to disaggregate data by treatment strategy and severity of ECOPD to enhance our understanding of these effects.

## 5. Conclusions

Our study on the COVID-19 pandemic’s impact on COPD management revealed a significant decrease in hospital admissions for AECOPD and an increase in mortality among hospitalized patients. These findings indicate the pandemic’s dual effects: patients’ reluctance to seek hospital care, likely due to fear of COVID-19, and potential challenges within healthcare systems that may have affected the quality of care for severe cases. The reduction in admissions suggests a shift in care-seeking behavior, while the rise in mortality points to issues in managing severe AECOPD during the pandemic. We recommend further research and targeted interventions to address care delivery for AECOPD and emphasize the importance of maintaining chronic disease care continuity during pandemics to prevent adverse outcomes.

## Figures and Tables

**Table 1:** Baseline characteristics for included studies.

Study	Location	Study Design	During the COVID-19 Pandemic				Before the COVID-19 Pandemic			
			Study Period	Sample Size	Male, *n* (%)	Age, Years	Study Period	Sample Size	Male, *n* (%)	Age, Years
Outcome: Mortality										
Argun Baris 2021 ^a^ [20]	Türkiye	Single center	1 March–30 April 2020	28	NR	NR	1 March–30 April 2018–2019	128	NR	NR
Chan 2020 [21]	China	Single center	January–March 2020	122	NR	NR	January–March 2015–2019	1385	NR	NR
Dang 2022 [22]	US	Multicenter (4626)	1 April 2020–30 September 2021	109,569	NR	NR	January–December 2019	167,997	NR	NR
de Miguel 2023 [23]	Spain	Multicenter	2020	51,396	NR	NR	2011–2019	607,033	NR	NR
Farrugia 2021 [24]	Malta	Single center	1 March–10 May 2020	119	89 (74.8)	70.9	1 March–10 May 2019	260	199 (76.5)	71.7
Fernandez Villar 2023 [25]	Spain	Multicenter	15 March 2020–30 June 2022	174	138 (79.3)	72 (63–77)	1 November 2019–14 March 2020	166	126 (75.9)	71 (63–77)
Ko 2023 [26]	China	Multicenter	January 2020 week 4–December 2020 week 4	11,065	9978 (90.3)	77.51 ± 10.16	January 2017 week 1–January 2020 week 3	67,628	59,463 (87.9)	77.72 ± 10.00
Kohli 2022 ^a^ [27]	US	Single center	1 April 2020–31 March 2021	68	NR	NR	1 January–30 December 2019	55	NR	NR
Poucineau 2022 [28]	France	Multicenter	1 January 2020–30 November 2021	138,725	NR	NR	1 January 2016–30 November 2019	427,165	NR	NR
Sarc 2021 [29]	Slovenia	Multicenter	March 2019–February 2021	867	543 (62.6)	71.8 ± 9.1	March 2015–February 2019	1538	986 (64.1)	71.0 ± 9.4
Sykes 2021 ^a^ [30]	UK	Multicenter	23 March–1 June 2020	666	39.5% ^c^	66.8 ± 15.2 ^c^	23 March–1 June 2018–2019	2167	38.2% ^c^	69.2 ^c^
Outcome: Hospitalization										
Acharya 2023 [31]	India	Single center	April 2020–August 2020	68	47 (69.1) ^d^	67.4 ± 7.1 ^d^	April 2019–August 2019	68	47 (69.1)	67.4 ± 7.1 ^d^
Bekele 2022 [32]	Hungary	Multicenter	15 March–30 May 2020	1000	409 (40.9)	NR	September 2019–January 2020	5408	2473 (45.7)	NR
Dang 2022 [22]	US	Multicenter	1 April 2020–30 September 2021	4,464,808	NR	NR	January–December 2019	3,983,950	NR	NR
Faria 2021 [33]	Portugal	Single center	March–July 2020	11	8/11(72.7)	72.0 ± 12.4	March–July, 2016–2019	114	114/153 (74.5)	68.7 ± 11.4 ^e^
González 2020 ^a^ [34]	Spain	Single center	1 March–31 May 2020	310 ^d^	257 (83) ^d^	67 ± 8 ^d^	1 March–31 May 2019	310 ^d^	257 (83) ^d^	67 ± 8 ^d^
Jaehn 2021 [35]	Germany	Multicenter	12 March–30 June 2020	41,353	50.5% ^c^	NR	12 March–30 June 2019	51,030	51.0% ^c^	NR
McAuley 2020 [36]	UK	Single center	15 March–30 April 2020	160 ^d^	88 (55) ^d^	67.3 ± 8.1 ^d^	15 March–30 April 2019	160 ^d^	88 (55) ^d^	67.3 ± 8.1 ^d^
Taylor 2021 ^a^ [37]	UK	Single center	23 March–23 June 2020	55	NR	NR	23 March–23 June 2019	72	NR	NR
Tokogoz Akyıl 2022 [38]	Türkiye	Single center	11 March–25 December 2020	256 ^d^	53 (21) ^d^	66 ± 10 ^d^	11 March–25 December 2019	256 ^d^	53 (21) ^d^	66 ± 10 ^d^
Wu 2023 [39]	China	Multicenter	January–December 2020	468 ^d^	306 (65.4)	75.3 ± 8.8	January 2018–December 2019	468 ^d^	306 (65.4)	75.3 ± 8.8
Zeng 2021 ^a^ [40]	China	Multicenter	1 January–31 May 2020	12,100	NR	NR	1 January–31 May 2017–2019	44,982	NR	NR

Values are shown as the mean ± standard deviation, median (interquartile range), or frequency (proportion). ^a^ These studies were conference abstracts or letters. ^b^ Details such as age and sex information for COPD were not provided for this cohort from the National Medicare Claim data, but information about the entire dataset is shown. ^c^ These data are for the total cohort, including COPD and other diseases. ^d^ These populations were the same patients in the cohort. ^e^ This was the value of the entire period, including the pandemic period. COPD, chronic obstructive pulmonary disease; COVID-19, coronavirus disease 2019; *n*, total size; NR, not reported; UK, United Kingdom; US, United States.

**Table 2:** Subgroup analysis for the in-hospital mortality and hospitalization of AECOPD during the COVID-19 pandemic compared with before the pandemic.

Characteristics	*n*	OR (95% CI)	*p*-Value for Heterogeneity	I^2^, %
Outcome: In-hospital mortality				
No. of hospitals conducting a study				
Multicenter	6	1.24 (1.17–1.31)	0.09	48
Single center	3	2.45 (1.40–4.26)	0.85	0
Sample size				
Large (≥1000 population)	5	1.24 (1.17–1.32)	0.09	51
Small (<1000 population)	4	2.18 (1.27–3.74)	0.35	8
Article type				
Original article	6	1.29 (1.18–1.42)	0.04	56
Letter or conference abstract	3	1.05 (0.41–2.70)	0.21	36
Outcome: Hospitalization				
No. of hospitals conducting a study				
Multicenter	5	0.65 (0.46–0.93)	<0.001	100
Single center	6	0.24 (0.06–0.97)	<0.001	95
Sample size				
Large (≥1000 population)	4	0.68 (0.44–1.05)	<0.001	100
Small (<1000 population)	7	0.27 (0.08–0.90)	<0.001	96
Article type				
Original article	8	0.33 (0.12–0.92)	<0.001	98
Letter or conference abstract	3	0.61 (0.25–1.53)	0.005	81
Same cohort				
Same patients	5	0.20 (0.04–0.90)	<0.001	97
Separate patients	6	0.70 (0.48–1.01)	<0.001	100

## Data Availability

The datasets generated during the study presented herein are available from the corresponding author upon reasonable request.

## Supplementary Materials

The following supporting information can be downloaded at: https://www.mdpi.com/article/10.3390/jpm14030296/s1, Table S1: Search strategy; Table S2: Quality assessments in individual studies by Newcastle–Ottawa tool; Figure S1: Forest plot of subgroup analysis for the in-hospital mortality of AECOPD during the COVID-19 pandemic compared with before the pandemic; Figure S2: Forest plot of subgroup analysis for the hospitalization of AECOPD during the COVID-19 pandemic compared with before the pandemic; Figure S3: Funnel plot. References [20,21,22,23,24,25,26,27,28,29,30,31,32,33,34,35,36,37,38,39,40] are cited in the supplementary materials.

## References

[1] Choi A., Kim H.Y., Cho A., Noh J., Park I., Chung H.S. (2021). Efficacy of a four-tier infection response system in the emergency department during the coronavirus disease—2019 outbreak. PLoS ONE.

[2] Blumenthal D., Fowler E.J., Abrams M., Collins S.R. (2020). COVID-19—Implications for the Health Care System. N. Engl. J. Med..

[3] Lee S.I., Kang S.B., Lee S.Y., Choi D.S. (2023). The effect of regional distribution of isolation rooms in emergency departments on ambulance travel time during the COVID-19 pandemic. Clin. Exp. Emerg. Med..

[4] Kim J.H., Ahn C., Namgung M. (2022). Comparative Evaluation of the Prognosis of Septic Shock Patients from before to after the Onset of the COVID-19 Pandemic: A Retrospective Single-Center Clinical Analysis. J. Pers. Med..

[5] Furnica C., Chistol R.O., Chiran D.A., Stan C.I., Sargu G.D., Girlescu N., Tinica G. (2022). The Impact of the Early COVID-19 Pandemic on ST-Segment Elevation Myocardial Infarction Presentation and Outcomes—A Systematic Review and Meta-Analysis. Diagnostics.

[6] Ishaque N., Butt A.J., Kamtchum-Tatuene J., Nomani A.Z., Razzaq S., Fatima N., Vekhande C., Nair R., Akhtar N., Khan K. (2022). Trends in Stroke Presentations before and during the COVID-19 Pandemic: A Meta-Analysis. J. Stroke.

[7] Lim Z.J., Ponnapa Reddy M., Afroz A., Billah B., Shekar K., Subramaniam A. (2020). Incidence and outcome of out-of-hospital cardiac arrests in the COVID-19 era: A systematic review and meta-analysis. Resuscitation.

[8] Kim J.H., Ahn C., Park Y., Won M. (2023). Comparison of out-of-hospital cardiac arrests during the COVID-19 pandemic with those before the pandemic: An updated systematic review and meta-analysis. Front. Public Health.

[9] Slagman A., Behringer W., Greiner F., Klein M., Weismann D., Erdmann B., Pigorsch M., Möckel M. (2020). Medical Emergencies during the COVID-19 Pandemic. Dtsch. Arztebl. Int..

[10] Dick L., Green J., Brown J., Kennedy E., Cassidy R., Othman S., Berlansky M. (2020). Changes in Emergency General Surgery during COVID-19 in Scotland: A Prospective Cohort Study. World J. Surg..

[11] Blanchette C.M., Dalal A.A., Mapel D. (2012). Changes in COPD demographics and costs over 20 years. J. Med. Econ..

[12] Agustí A., Celli B.R., Criner G.J., Halpin D., Anzueto A., Barnes P., Bourbeau J., Han M.K., Martinez F.J., Montes de Oca M. (2023). Global Initiative for Chronic Obstructive Lung Disease 2023 Report: GOLD Executive Summary. Eur. Respir. J..

[13] Santibáñez M., Garrastazu R., Ruiz-Nuñez M., Helguera J.M., Arenal S., Bonnardeux C., León C., García-Rivero J.L. (2016). Predictors of Hospitalized Exacerbations and Mortality in Chronic Obstructive Pulmonary Disease. PLoS ONE.

[14] Halpin D.M., Miravitlles M., Metzdorf N., Celli B. (2017). Impact and prevention of severe exacerbations of COPD: A review of the evidence. Int. J. Chronic Obstr. Pulm. Dis..

[15] Pappe E., Hammerich R., Saccomanno J., Sgarbossa T., Pohrt A., Schmidt B., Grah C., Eisenmann S., Holland A., Eggeling S. (2023). Impact of Coronavirus Disease 2019 on Hospital Admissions, Health Status, and Behavioral Changes of Patients with COPD. Chronic Obstr. Pulm. Dis..

[16] Hutton B., Salanti G., Caldwell D.M., Chaimani A., Schmid C.H., Cameron C., Ioannidis J.P., Straus S., Thorlund K., Jansen J.P. (2015). The PRISMA extension statement for reporting of systematic reviews incorporating network meta-analyses of health care interventions: Checklist and explanations. Ann. Intern. Med..

[17] Stroup D.F., Berlin J.A., Morton S.C., Olkin I., Williamson G.D., Rennie D., Moher D., Becker B.J., Sipe T.A., Thacker S.B. (2000). Meta-analysis of observational studies in epidemiology: A proposal for reporting. Meta-analysis Of Observational Studies in Epidemiology (MOOSE) group. JAMA.

[18] Stang A. (2010). Critical evaluation of the Newcastle-Ottawa scale for the assessment of the quality of nonrandomized studies in meta-analyses. Eur. J. Epidemiol..

[19] Higgins J.P., Thompson S.G. (2002). Quantifying heterogeneity in a meta-analysis. Stat. Med..

[20] Argun Baris S., Boyaci H., Kaya H., Basyigit I. (2021). Reduced Rate of Hospital Admission for Exacerbation of COPD and Asthma during COVID-19 Pandemic. Turk. Thorac. J..

[21] Chan K.P.F., Ma T.F., Kwok W.C., Leung J.K.C., Chiang K.Y., Ho J.C.M., Lam D.C.L., Tam T.C.C., Ip M.S.M., Ho P.L. (2020). Significant reduction in hospital admissions for acute exacerbation of chronic obstructive pulmonary disease in Hong Kong during coronavirus disease 2019 pandemic. Respir. Med..

[22] Dang A., Thakker R., Li S., Hommel E., Mehta H.B., Goodwin J.S. (2022). Hospitalizations and Mortality from Non-SARS-CoV-2 Causes among Medicare Beneficiaries at US Hospitals during the SARS-CoV-2 Pandemic. JAMA Netw. Open.

[23] de Miguel-Diez J., Lopez-de-Andres A., Jimenez-Garcia R., Hernández-Barrera V., Carabantes-Alarcon D., Zamorano-Leon J.J., Omaña-Palanco R., González-Barcala F.J., Cuadrado-Corrales N. (2023). Trends in prevalence and the effects on hospital outcomes of dementia in patients hospitalized with acute COPD exacerbation. Respir. Med..

[24] Farrugia Y., Spiteri Meilak B.P., Grech N., Asciak R., Camilleri L., Montefort S., Zammit C. (2021). The Impact of COVID-19 on Hospitalised COPD Exacerbations in Malta. Pulm. Med..

[25] Fernández Villar A., Golpe Gómez R., González Montaos A., Fernández García S., Pazos Area L., Priegue Carrera A., Ruano Raviña A., Represas Represas C. (2023). The impact of the SARS-CoV-2 pandemic on the demographic, clinical and social profiles of patients admitted to the Pneumology Department for a COPD exacerbation. PLoS ONE.

[26] Ko F.W.S., Lau L.H.S., Ng S.S., Yip T.C.F., Wong G.L.H., Chan K.P., Chan T.O., Hui D.S.C. (2023). Respiratory admissions before and during the COVID-19 pandemic with mediation analysis of air pollutants, mask-wearing and influenza rates. Respirology.

[27] Kohli A., Alnafoosi Z., White P., Abdulfattah O. (2022). Impact of the COVID-19 pandemic on hospital admission rate, length of stay, and mortalityt rate for patients with COPD exaverbation: A retrospective study. Chest.

[28] Poucineau J., Delory T., Lapidus N., Hejblum G., Chouaïd C., Le Cœur S., Khlat M. (2022). Hospital admissions and mortality for acute exacerbations of COPD during the COVID-19 pandemic: A nationwide study in France. Front. Med..

[29] Sarc I., Lotric Dolinar A., Morgan T., Sambt J., Ziherl K., Gavric D., Selb J., Rozman A., Dosenovic Bonca P. (2022). Mortality, seasonal variation, and susceptibility to acute exacerbation of COPD in the pandemic year: A nationwide population study. Ther. Adv. Respir. Dis..

[30] Sykes D.L., Faruqi S., Holdsworth L., Crooks M.G. (2021). Impact of COVID-19 on COPD and asthma admissions, and the pandemic from a patient’s perspective. ERJ Open Res..

[31] Acharya V.K., Sharma D.K., Kamath S.K., Shreenivasa A., Unnikrishnan B., Holla R., Gautham M., Rathi P., Mendonca J. (2023). Impact of COVID-19 Pandemic on the Exacerbation Rates in COPD Patients in Southern India—A Potential Role for Community Mitigations Measures. Int. J. Chronic Obstr. Pulm. Dis..

[32] Bekele B.B., Alhaffar B.A., Wasnik R.N., Sándor J. (2022). The Effect of the COVID-19 Pandemic on the Social Inequalities of Health Care Use in Hungary: A Nationally Representative Cross-Sectional Study. Int. J. Environ. Res. Public Health.

[33] Faria N., Costa M.I., Gomes J., Sucena M. (2021). Reduction of Severe Exacerbations of COPD during COVID-19 Pandemic in Portugal: A Protective Role of Face Masks?. COPD J. Chronic Obstr. Pulm. Dis..

[34] González J., Moncusí-Moix A., Benitez I.D., Santisteve S., Monge A., Fontiveros M.A., Carmona P., Torres G., Barbé F., de Batlle J. (2021). Clinical Consequences of COVID-19 Lockdown in Patients with COPD: Results of a Pre-Post Study in Spain. Chest.

[35] Jaehn P., Holmberg C., Uhlenbrock G., Pohl A., Finkenzeller T., Pawlik M.T., Quack I., Ernstberger A., Rockmann F., Schreyer A.G. (2021). Differential trends of admissions in accident and emergency departments during the COVID-19 pandemic in Germany. BMC Emerg. Med..

[36] McAuley H., Hadley K., Elneima O., Brightling C.E., Evans R.A., Steiner M.C., Greening N.J. (2021). COPD in the time of COVID-19: An analysis of acute exacerbations and reported behavioural changes in patients with COPD. ERJ Open Res..

[37] Taylor A., Simpson T., Joseph T. (2021). P32 Reduction in the rate of acute exacerbations of COPD and asthma during the COVID-19 pandemic. Thorax.

[38] Tokgöz Akyıl F., Tural Önür S., Sökücü S., Abalı H., Boyracı N., Çayır E., Altın S. (2022). Lifestyle Changes and Exacerbation Frequency of COPD in Times of the Pandemic. Turk. Thorac. J..

[39] Wu T.T., Jiang Y.Q., Zhao B.F., Si F.L., Wu P., Wang H.Y., Sheng C.F., Xu X., Li F., Zhang J. (2023). Real-World COPD Management Over 3 Years at the Community Health Service Center of Shanghai during the COVID-19 Pandemic in China. Int. J. Chronic Obstr. Pulm. Dis..

[40] Zeng Y., Zhou Z., Chen P. (2021). Policy during coronavirus disease 2019 (COVID-19) pandemic: A protector for acute exacerbation of COPD (AECOPD) patients?. J. Thorac. Dis..

[41] Tan J.Y., Conceicao E.P., Wee L.E., Sim X.Y.J., Venkatachalam I. (2021). COVID-19 public health measures: A reduction in hospital admissions for COPD exacerbations. Thorax.

[42] Smulowitz P.B., O’Malley A.J., Khidir H., Zaborski L., McWilliams J.M., Landon B.E. (2021). National Trends in ED Visits, Hospital Admissions, and Mortality for Medicare Patients during the COVID-19 Pandemic. Health Aff..

[43] Birkmeyer J.D., Barnato A., Birkmeyer N., Bessler R., Skinner J. (2020). The Impact of The COVID-19 Pandemic on Hospital Admissions in the United States. Health Aff..

[44] Alsallakh M.A., Sivakumaran S., Kennedy S., Vasileiou E., Lyons R.A., Robertson C., Sheikh A., Davies G.A. (2021). Impact of COVID-19 lockdown on the incidence and mortality of acute exacerbations of chronic obstructive pulmonary disease: National interrupted time series analyses for Scotland and Wales. BMC Med..

[45] So J.Y., O’Hara N.N., Kenaa B., Williams J.G., deBorja C.L., Slejko J.F., Zafari Z., Sokolow M., Zimand P., Deming M. (2021). Population Decline in COPD Admissions during the COVID-19 Pandemic Associated with Lower Burden of Community Respiratory Viral Infections. Am. J. Med..

[46] Sigala I., Giannakas T., Giannakoulis V.G., Zervas E., Brinia A., Gianiou N., Asimakos A., Dima E., Kalomenidis I., Katsaounou P. (2021). Effect of COVID-19-Related Lockdown οn Hospital Admissions for Asthma and COPD Exacerbations: Associations with Air Pollution and Patient Characteristics. J. Pers. Med..

[47] Simons S.O., Hurst J.R., Miravitlles M., Franssen F.M.E., Janssen D.J.A., Papi A., Duiverman M.L., Kerstjens H.A.M. (2020). Caring for patients with COPD and COVID-19: A viewpoint to spark discussion. Thorax.

[48] Higham A., Mathioudakis A., Vestbo J., Singh D. (2020). COVID-19 and COPD: A narrative review of the basic science and clinical outcomes. Eur. Respir. Rev..

[49] Janke A.T., Mei H., Rothenberg C., Becher R.D., Lin Z., Venkatesh A.K. (2021). Analysis of Hospital Resource Availability and COVID-19 Mortality across the United States. J. Hosp. Med..

[50] Madawala S., Quach A., Lim J.Y., Varatharaj S., Perera B., Osadnik C., Barton C. (2023). Healthcare experience of adults with COPD during the COVID-19 pandemic: A rapid review of international literature. BMJ Open Respir. Res..

[51] Hacker K.A., Briss P.A., Richardson L., Wright J., Petersen R. (2021). COVID-19 and Chronic Disease: The Impact Now and in the Future. Prev. Chronic Dis..

[52] Fekadu G., Bekele F., Tolossa T., Fetensa G., Turi E., Getachew M., Abdisa E., Assefa L., Afeta M., Demisew W. (2021). Impact of COVID-19 pandemic on chronic diseases care follow-up and current perspectives in low resource settings: A narrative review. Int. J. Physiol. Pathophysiol. Pharmacol..

[53] Van den Bulck S., Crèvecoeur J., Aertgeerts B., Delvaux N., Neyens T., Van Pottelbergh G., Coursier P., Vaes B. (2022). The impact of the COVID-19 pandemic on the incidence of diseases and the provision of primary care: A registry-based study. PLoS ONE.

[54] Danhieux K., Buffel V., Pairon A., Benkheil A., Remmen R., Wouters E., van Olmen J. (2020). The impact of COVID-19 on chronic care according to providers: A qualitative study among primary care practices in Belgium. BMC Fam. Pract..

[55] Halpin D.M.G., Vogelmeier C.F., Agusti A. (2021). COVID-19 and COPD: Lessons beyond the pandemic. Am. J. Physiol. Lung Cell Mol. Physiol..

[56] Houchen-Wolloff L., Ward S., Chaplin E., Gardiner N., Singh S.J. (2021). P65 Remote delivery options for self-management programmes for patients with COPD during the COVID-19 pandemic. Uptake, completion and clinical outcomes. Break. Barriers Pulm. Rehabil. Physiother..

