# Supplementary material for: Chronic Obstructive Pulmonary Disease Mortality and Hospitalization during the COVID-19 Pandemic Compared with before the Pandemic: A Systematic Review and Meta-Analysis
Source: J Pers Med. 2024 Mar 10;14(3):296. doi: 10.3390/jpm14030296 (PMC10970825; doi:10.3390/jpm14030296)
Supplement: Supplementary file 1 [file jpm-14-00296-s001.zip › jpm-2905993-supplementary.pdf]

Chronic Obstructive Pulmonary Disease Mortality and Hospitalization During the COVID-19 Pandemic Compared with before the Pandemic: A Systematic Review and Meta-analysis

Chiwon Ahn[1] and Yeonkyung Park[2,3,*]

Supplemental Material

Supplementary Table S1. Search strategy

Supplementary Table S2. Quality assessments in individual studies by Newcastle-Ottawa tool

Supplementary Figure S1. Forest plot of subgroup analysis for the in-hospital mortality of AECOPD during the COVID-19 pandemic compared with before the pandemic

Supplementary Figure S2. Forest plot of subgroup analysis for the hospitalization of AECOPD during the COVID-19 pandemic compared with before the pandemic

Supplementary Figure S3. Funnel plot

Supplementary Table S1. Search strategy (searched on 31 December, 2023)

Database	Search term	N
Medline	Coronavirus Infections/ OR Coronavirus/ OR coronavirus.ti,ab. OR COVID-19.ti,ab. OR novel coronavirus 2019.ti,ab. OR SARS-CoV.ti,ab. OR SARS-CoV-2.ti,ab. OR 2019-nCoV.ti,ab. OR sars covid 19.ti,ab. OR sars-covid-2.ti,ab. OR severe acute respiratory syndrome.mp.<br>AND<br>Pulmonary Disease, Chronic Obstructive/ OR (obstructive adj3 (lung or pulmonary or respiratory or bronchopulmonary)).mp. OR (COAD or COBD or COPD).mp. OR Emphysema/<br>AND<br>Patient Admission/ OR Hospitalization/ OR exacerbation*.mp. OR exacerbate*.mp.<br>AND<br>Limit to yr="2019-Current"	1,551
Embase	'coronavirus infection'/exp OR 'coronavirus infection' OR 'covid 19' OR coronavirus OR "novel AND coronavirus AND 2019" OR 'sars cov' OR 'sars cov 2' OR '2019 ncov' OR "sars AND covid AND 19" OR "coronavirus related coronavirus related"<br>AND<br>'chronic obstructive lung disease' OR coad OR cobd OR copd OR 'emphysema'<br>AND<br>'hospital admission' OR 'hospitalization' OR 'exacerbation' OR exacerbate*<br>AND<br>Limit to yr="2019-Current"	2,977
Cochrane library	('COVID-19' OR 'coronavirus' OR 'severe acute respiratory syndrome coronavirus' OR 'SARS-CoV')<br>AND<br>('COPD' OR 'chronic obstructive pulmonary disease' OR 'emphysema')	161

Total	4,689

AECOPD, acute exacerbation of chronic obstructive pulmonary disease; COPD, chronic obstructive pulmonary disease; COVID-19, coronavirus disease 2019;

Supplementary Table S2. Quality assessments in individual studies by Newcastle-Ottawa tool

Study	Selection				Comparability	Outcome / Exposure			Total
	Representativeness of the exposed cohort	Selection of the non-exposed cohort	Ascertainment of exposure	Demonstration that outcome of interest was not present at the start of the study	Comparability of cohorts based on the design or analysis controlled for confounders	Assessment of outcome	Was follow-up long enough for outcomes to occur	Adequacy of follow-up of cohorts	
Argun Baris 2021[a]	★	★	★	★	-	★	-	-	5
Chan 2020	★	★	★	★	-	★	-	-	5
Dang 2022	★	★	★	★	★★	★	-	-	7
de Miguel 2023	★	★	★	★	★★	★	-	-	7
Farrugia 2021	★	★	★	★	★★	★	-	-	7
Fernandez Villar 2023	★	★	★	★	★★	★	-	-	7
Ko 2023	★	★	★	★	-	★	-	-	5
Kohli 2022[a]	★	★	★	★	-	★	-	-	5
Poucineau 2022	★	★	★	★	-	★	-	-	5
Sarc 2021	★	★	★	★	★★	★	-	-	7
Sykes 2021[a]	★	★	★	★	-	★	-	-	5
Acharya 2023									
Bekele 2022	★	★	★	★	★★	★	-	-	7
Faria 2021	★	★	★	★	-	★	-	-	5
González 2020[a]	★	★	★	★	-	★	-	-	5
Jaehn 2021	★	★	★	★	★★	★	-	-	7
McAuley 2020	★	★	★	★	-	★	-	-	5

Taylor 2021[a]	★	★	★	★	-	★	-	-	5
Tokgöz Akyıl 2022	★	★	★	★	★★	★	-	-	7
Wu 2023	★	★	★	★	-	★	-	-	5
Zeng 2021[a]	★	★	★	★	-	★	-	-	5

[a]These studies were conference abstracts or letters.

Supplementary Figure S1. Forest plot of subgroup analysis for the in-hospital mortality of AECOPD during the COVID-19 pandemic compared with before the pandemic. (A) No. of hospitals conducting a study, (B) Sample size, and (C) Article type.

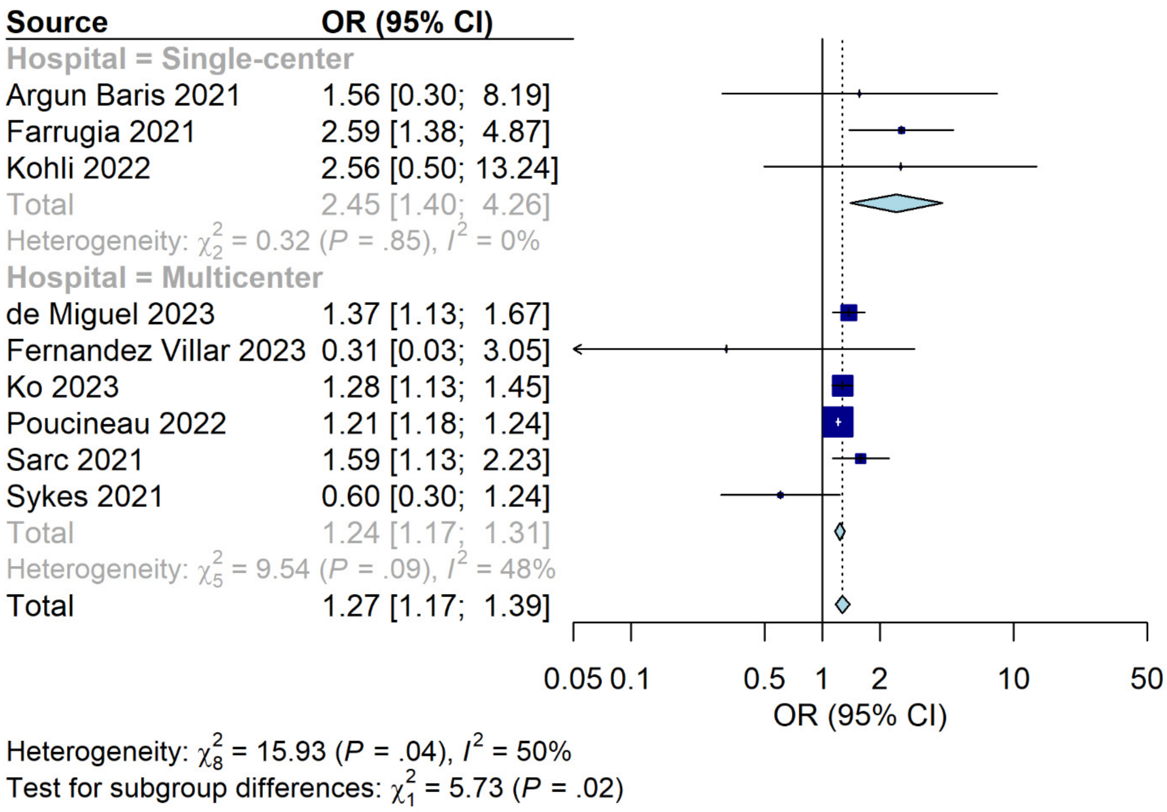

(A) No. of hospitals conducting a study

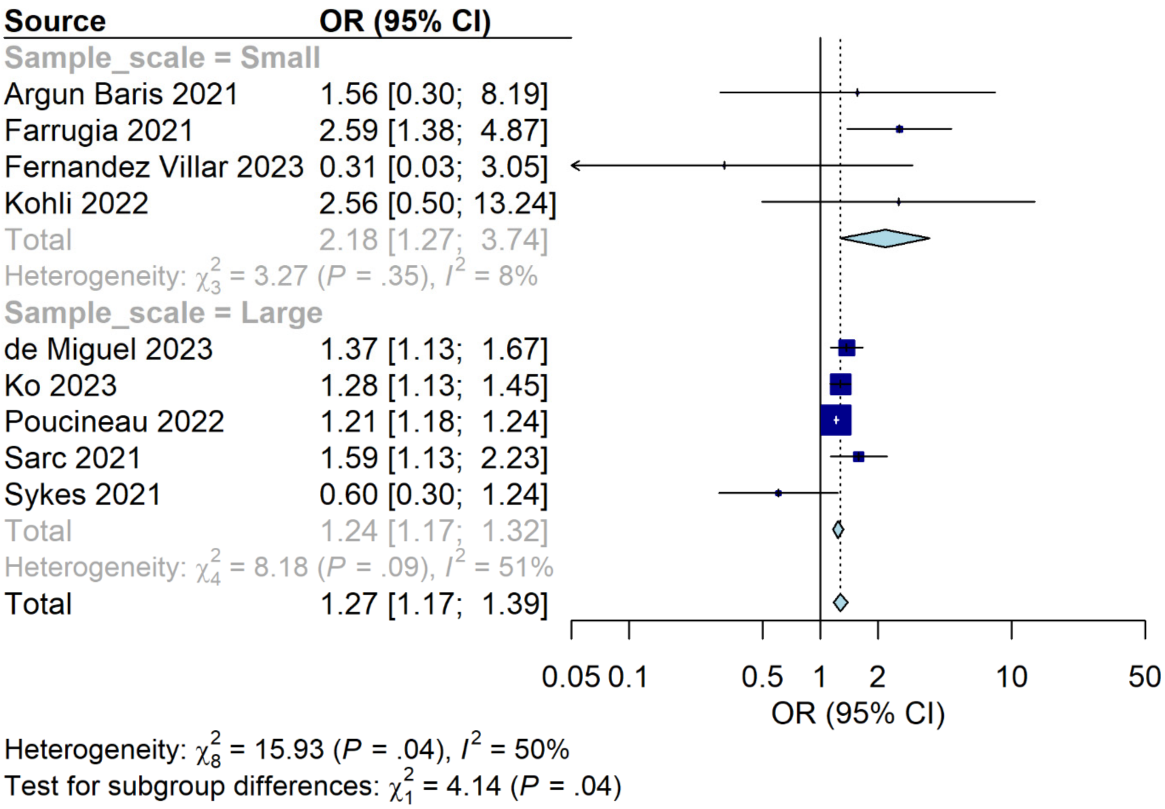

(B) Sample size

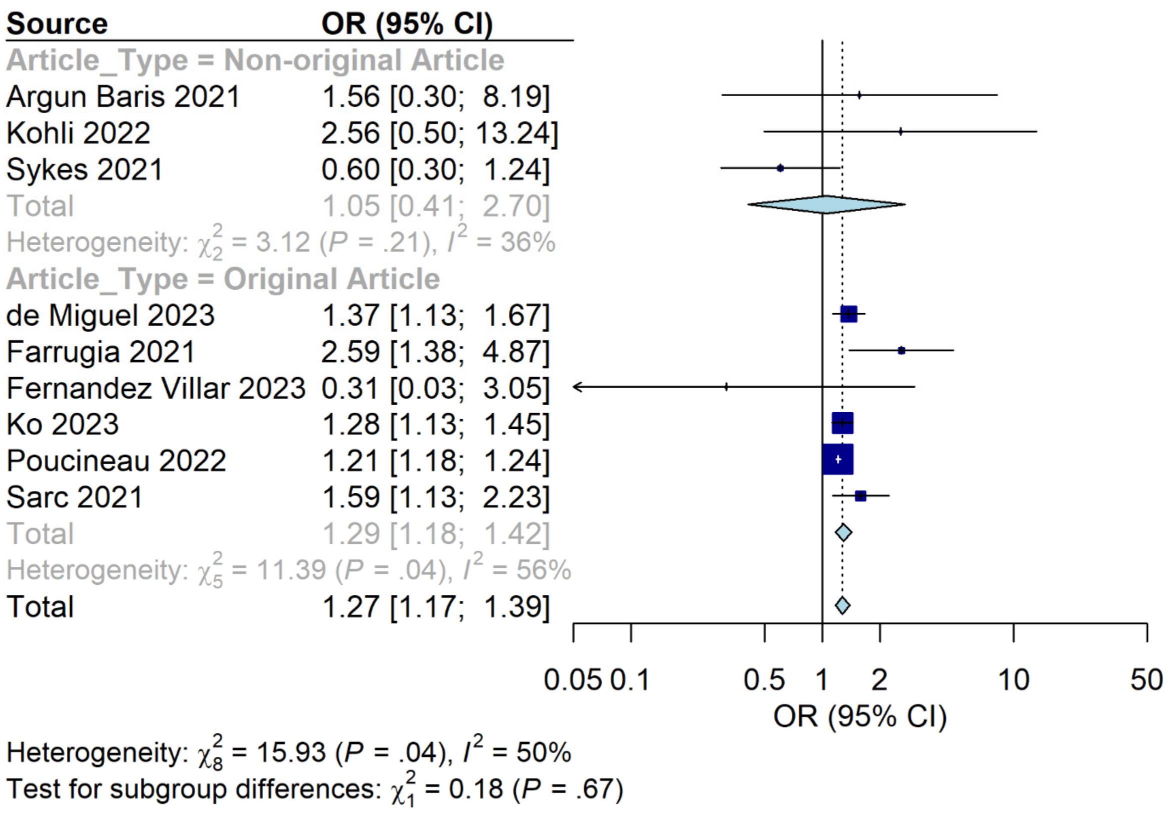

(C) Article type

Supplementary Figure S2. Forest plot of subgroup analysis for the hospitalization of AECOPD during the COVID-19 pandemic compared with before the pandemic. (A) No. of hospitals conducting a study, (B) Sample size, (C) Article type, and (D) Sameness cohort.

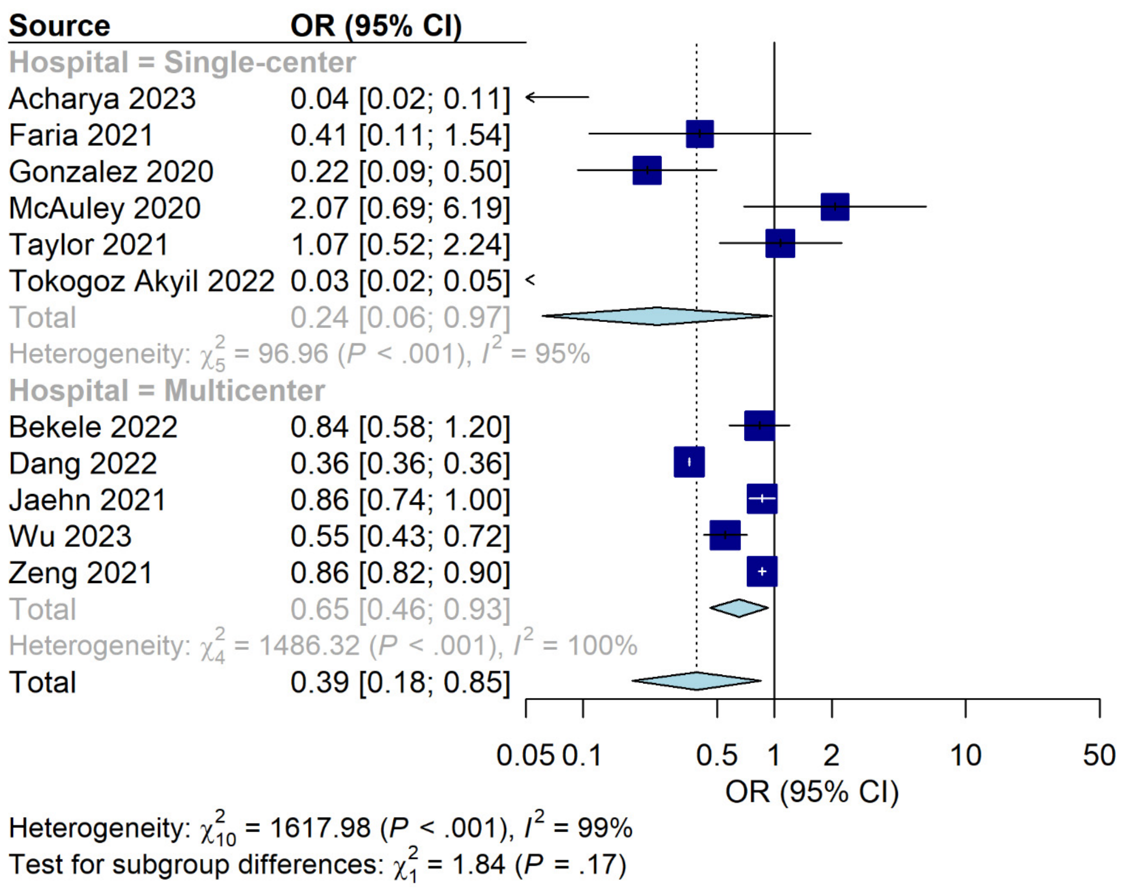

(A) No. of hospitals conducting a study

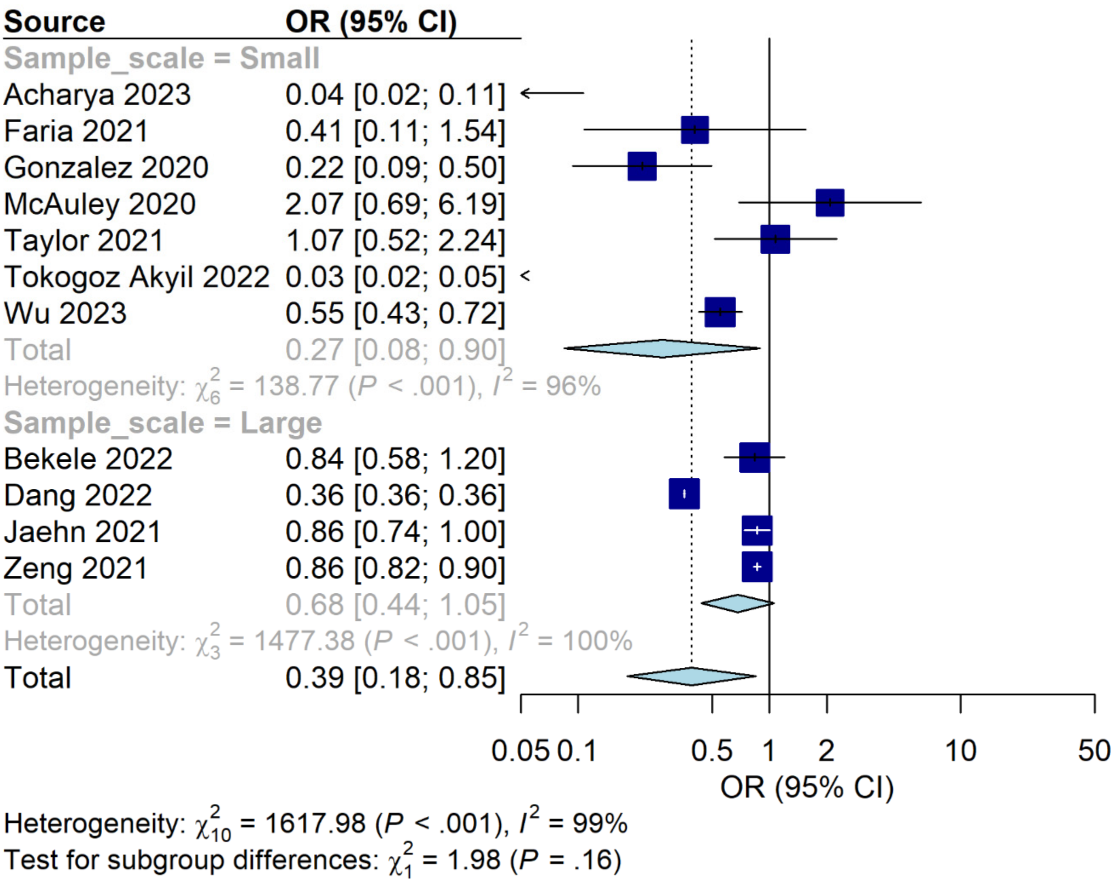

(B) Sample size

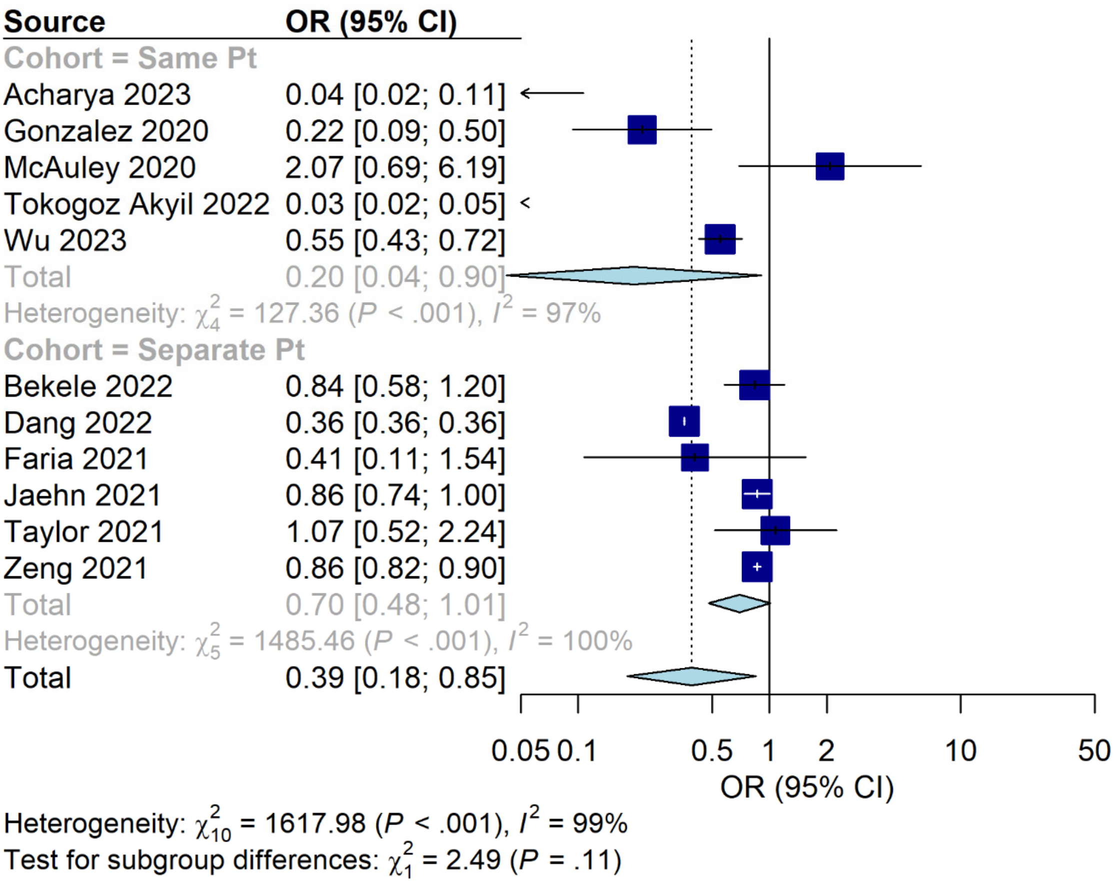

(C) Article type

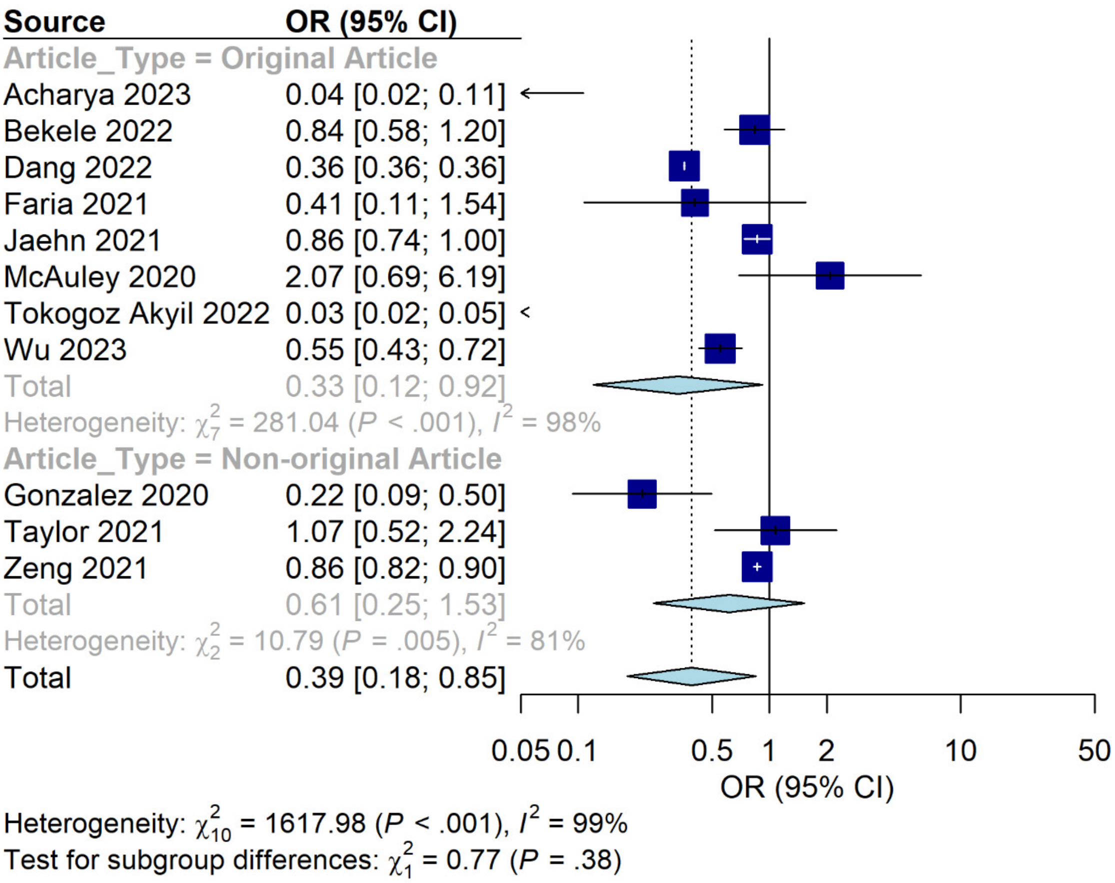

(D) Sameness cohort.

Supplementary Figure S3. Funnel plot. (A) Mortality and (B) Hospitalization.

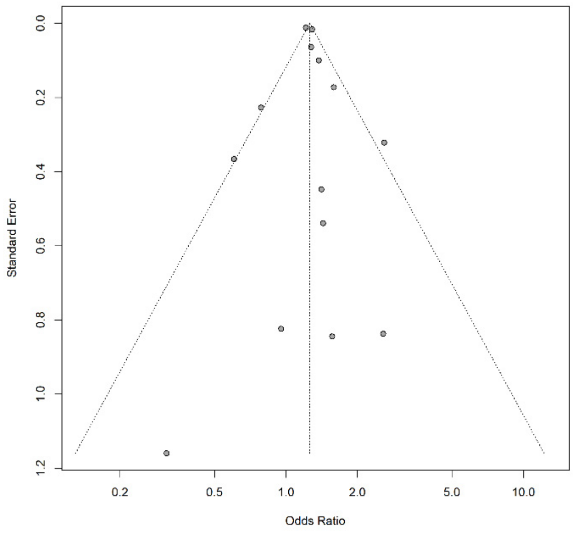

(A) Mortality

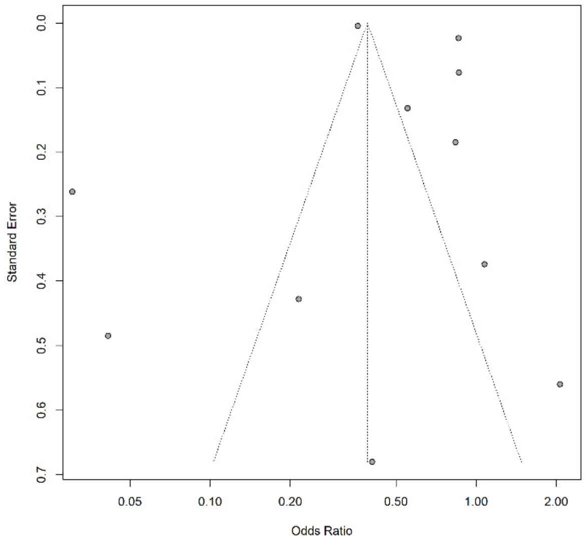

(B) Hospitalization.
