# Supplementary material for: Chronic Obstructive Pulmonary Disease Mortality and Hospitalization during the COVID-19 Pandemic Compared with before the Pandemic: A Systematic Review and Meta-Analysis
Source: J Pers Med. 2024 Mar 10;14(3):296. doi: 10.3390/jpm14030296 (PMC10970825; doi:10.3390/jpm14030296)
Supplement: Supplementary file 1 [file jpm-14-00296-s001.zip › supplemental_materials.docx]

# Chronic Obstructive Pulmonary Disease Mortality and Hospitalization During the COVID-19 Pandemic Compared with before the Pandemic: A Systematic Review and Meta-analysis

Chiwon Ahn^1^ and Yeonkyung Park^2,3,*^

**Supplemental Material**

**Supplementary Table S1.** Search strategy

**Supplementary Table S2.** Quality assessments in individual studies by Newcastle-Ottawa tool

**Supplementary Figure S1.** Forest plot of subgroup analysis for the in-hospital mortality of AECOPD during the COVID-19 pandemic compared with before the pandemic

**Supplementary Figure S2.** Forest plot of subgroup analysis for the hospitalization of AECOPD during the COVID-19 pandemic compared with before the pandemic

**Supplementary Figure S3.** Funnel plot

**Supplementary Table S1.** Search strategy (searched on 31 December, 2023)

| **Database** | **Search term** | **N** |
| --- | --- | --- |
| Medline | Coronavirus Infections/ OR Coronavirus/ OR coronavirus.ti,ab. OR COVID-19.ti,ab. OR novel coronavirus 2019.ti,ab. OR SARS-CoV.ti,ab. OR SARS-CoV-2.ti,ab. OR 2019-nCoV.ti,ab. OR sars covid 19.ti,ab. OR sars-covid-2.ti,ab. OR severe acute respiratory syndrome.mp.  AND  Pulmonary Disease, Chronic Obstructive/ OR (obstructive adj3 (lung or pulmonary or respiratory or bronchopulmonary)).mp. OR (COAD or COBD or COPD).mp. OR Emphysema/  AND  Patient Admission/ OR Hospitalization/ OR exacerbation*.mp. OR exacerbate*.mp.  AND  Limit to yr=“2019-Current” | 1,551 |
| Embase | 'coronavirus infection'/exp OR 'coronavirus infection' OR 'covid 19' OR coronavirus OR “novel AND coronavirus AND 2019” OR 'sars cov' OR 'sars cov 2' OR '2019 ncov' OR “sars AND covid AND 19” OR “coronavirus related coronavirus related”  AND  'chronic obstructive lung disease' OR coad OR cobd OR copd OR 'emphysema'  AND  'hospital admission' OR 'hospitalization' OR 'exacerbation' OR exacerbate*  AND  Limit to yr=“2019-Current” | 2,977 |
| Cochrane library | (‘COVID-19’ OR ‘coronavirus’ OR ‘severe acute respiratory syndrome coronavirus’ OR ‘SARS-CoV’)  AND  (‘COPD’ OR ‘chronic obstructive pulmonary disease’ OR ‘emphysema’) | 161 |
| Total |  | 4,689 |

AECOPD, acute exacerbation of chronic obstructive pulmonary disease; COPD, chronic obstructive pulmonary disease; COVID-19, coronavirus disease 2019;

**Supplementary Table S2.** Quality assessments in individual studies by Newcastle-Ottawa tool

| Study | Selection | | | | Comparability | Outcome / Exposure | | | Total |
| --- | --- | --- | --- | --- | --- | --- | --- | --- | --- |
|  | Representativeness of the exposed cohort | Selection of the non-exposed cohort | Ascertainment of exposure | Demonstration that outcome of interest was not present at the start of the study | Comparability of cohorts based on the design or analysis controlled for confounders | Assessment of outcome | Was follow-up long enough for outcomes to occur | Adequacy of follow-up of cohorts |  |
| Argun Baris 2021^a^ | ★ | ★ | ★ | ★ | - | ★ | - | - | 5 |
| Chan 2020 | ★ | ★ | ★ | ★ | - | ★ | - | - | 5 |
| Dang 2022 | ★ | ★ | ★ | ★ | ★★ | ★ | - | - | 7 |
| de Miguel 2023 | ★ | ★ | ★ | ★ | ★★ | ★ | - | - | 7 |
| Farrugia 2021 | ★ | ★ | ★ | ★ | ★★ | ★ | - | - | 7 |
| Fernandez Villar 2023 | ★ | ★ | ★ | ★ | ★★ | ★ | - | - | 7 |
| Ko 2023 | ★ | ★ | ★ | ★ | - | ★ | - | - | 5 |
| Kohli 2022^a^ | ★ | ★ | ★ | ★ | - | ★ | - | - | 5 |
| Poucineau 2022 | ★ | ★ | ★ | ★ | - | ★ | - | - | 5 |
| Sarc 2021 | ★ | ★ | ★ | ★ | ★★ | ★ | - | - | 7 |
| Sykes 2021^a^ | ★ | ★ | ★ | ★ | - | ★ | - | - | 5 |
| Acharya 2023 |  |  |  |  |  |  |  |  |  |
| Bekele 2022 | ★ | ★ | ★ | ★ | ★★ | ★ | - | - | 7 |
| Faria 2021 | ★ | ★ | ★ | ★ | - | ★ | - | - | 5 |
| González 2020^a^ | ★ | ★ | ★ | ★ | - | ★ | - | - | 5 |
| Jaehn 2021 | ★ | ★ | ★ | ★ | ★★ | ★ | - | - | 7 |
| McAuley 2020 | ★ | ★ | ★ | ★ | - | ★ | - | - | 5 |
| Taylor 2021^a^ | ★ | ★ | ★ | ★ | - | ★ | - | - | 5 |
| Tokgöz Akyıl 2022 | ★ | ★ | ★ | ★ | ★★ | ★ | - | - | 7 |
| Wu 2023 | ★ | ★ | ★ | ★ | - | ★ | - | - | 5 |
| Zeng 2021^a^ | ★ | ★ | ★ | ★ | - | ★ | - | - | 5 |

^a^These studies were conference abstracts or letters.

**Supplementary Figure S1.** Forest plot of subgroup analysis for the in-hospital mortality of AECOPD during the COVID-19 pandemic compared with before the pandemic. (A) No. of hospitals conducting a study, (B) Sample size, and (C) Article type.


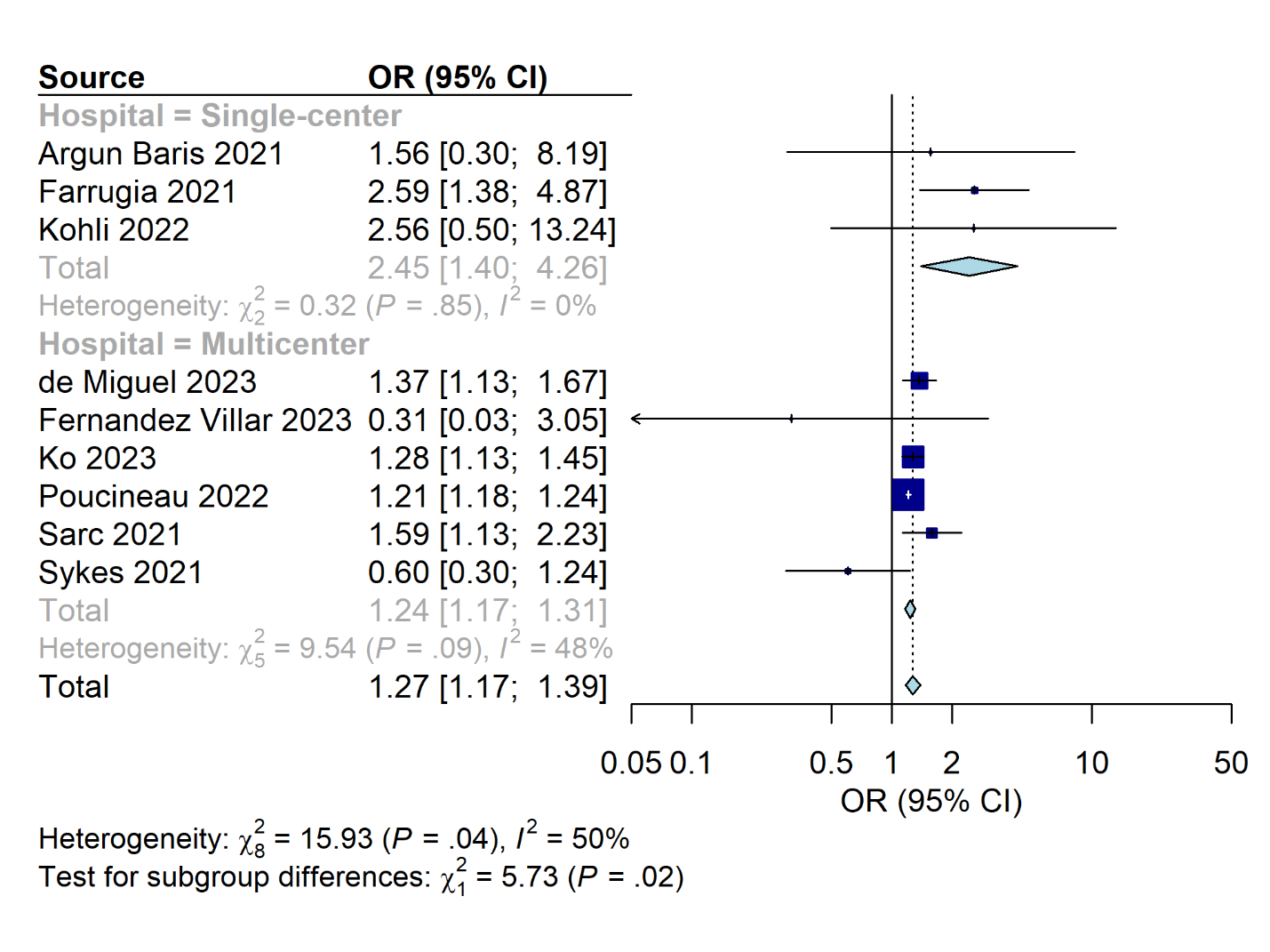


(A) No. of hospitals conducting a study


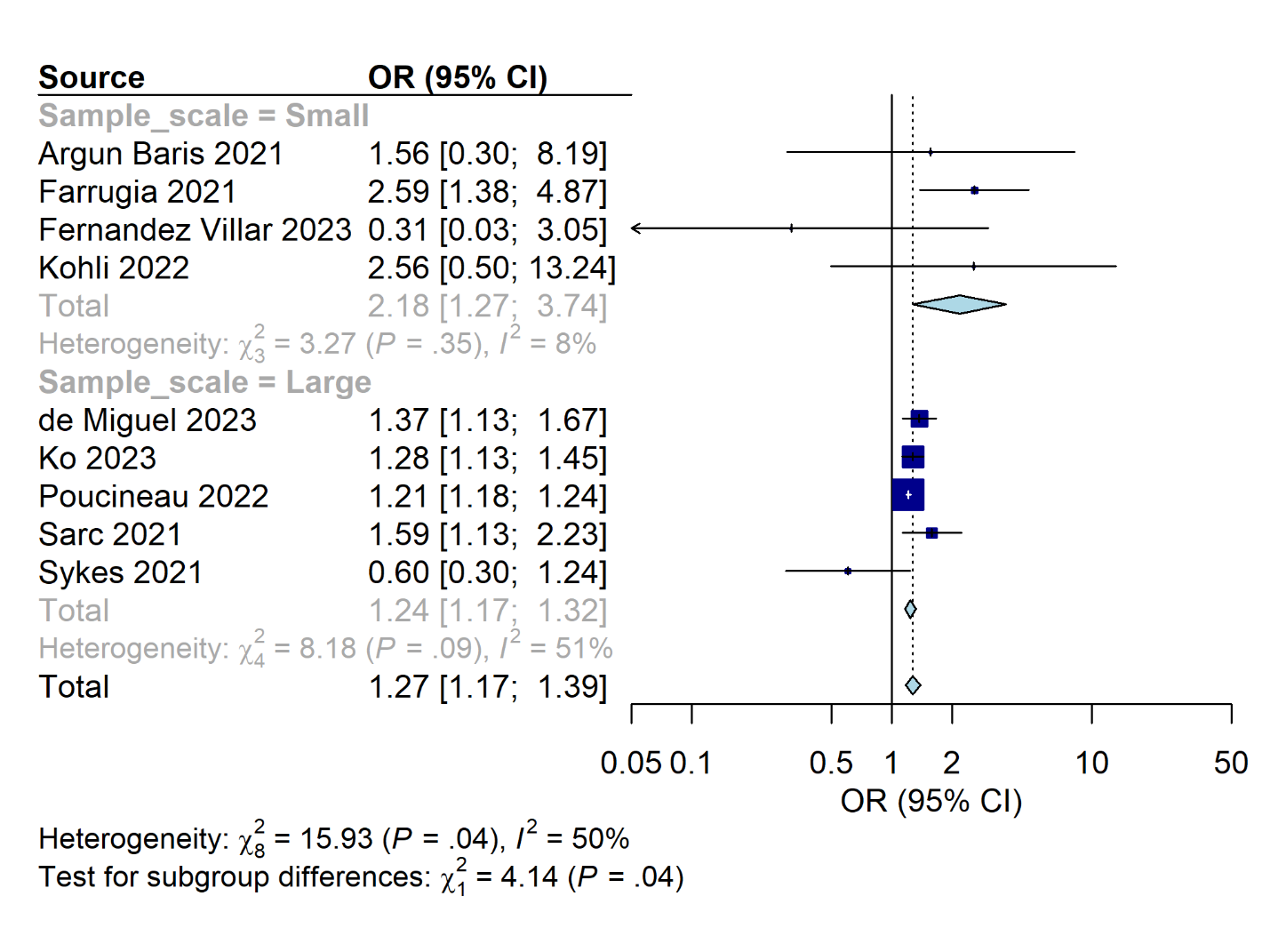


(B) Sample size


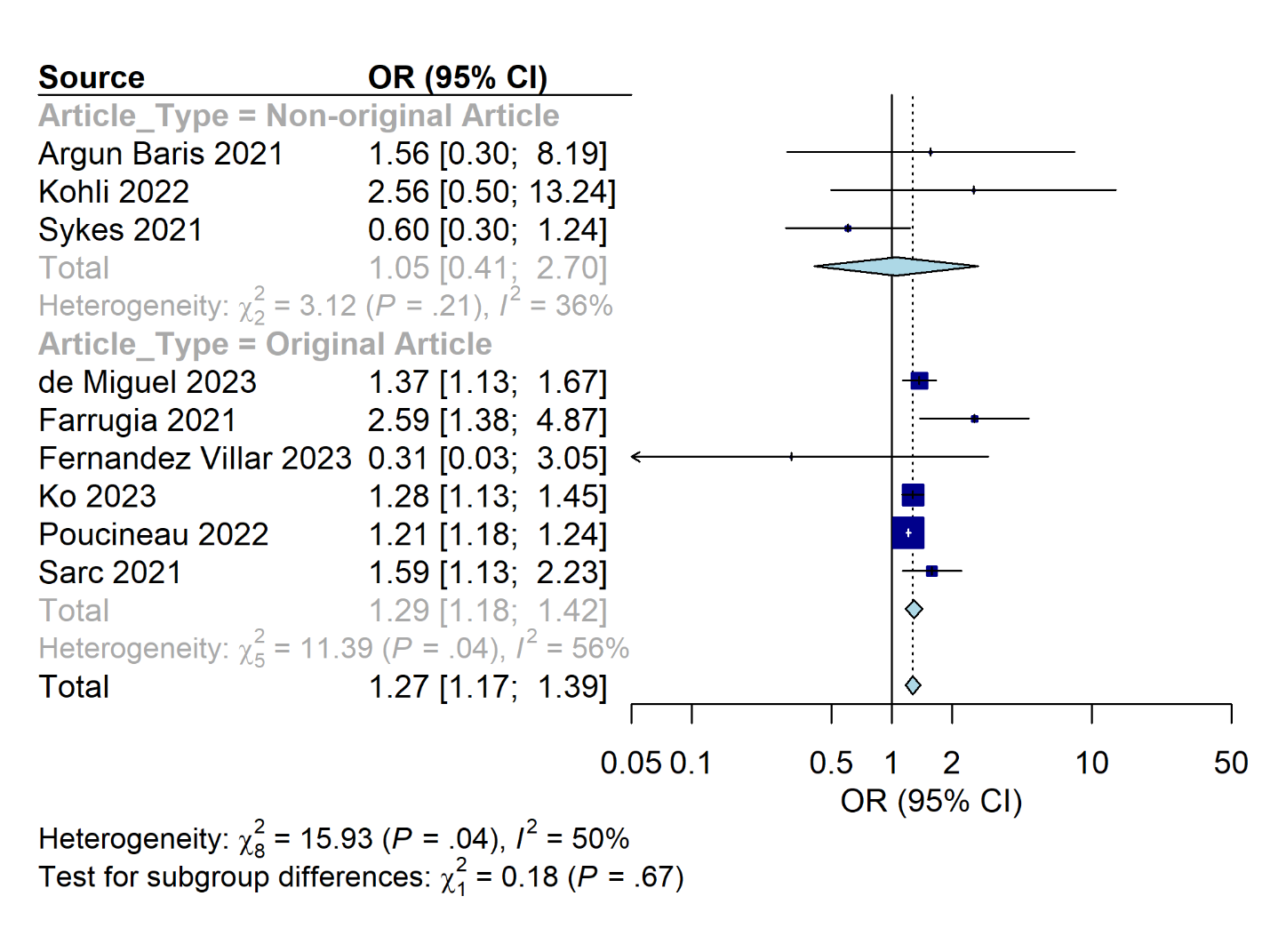


(C) Article type

**Supplementary Figure S2.** Forest plot of subgroup analysis for the hospitalization of AECOPD during the COVID-19 pandemic compared with before the pandemic. (A) No. of hospitals conducting a study, (B) Sample size, (C) Article type, and (D) Sameness cohort.


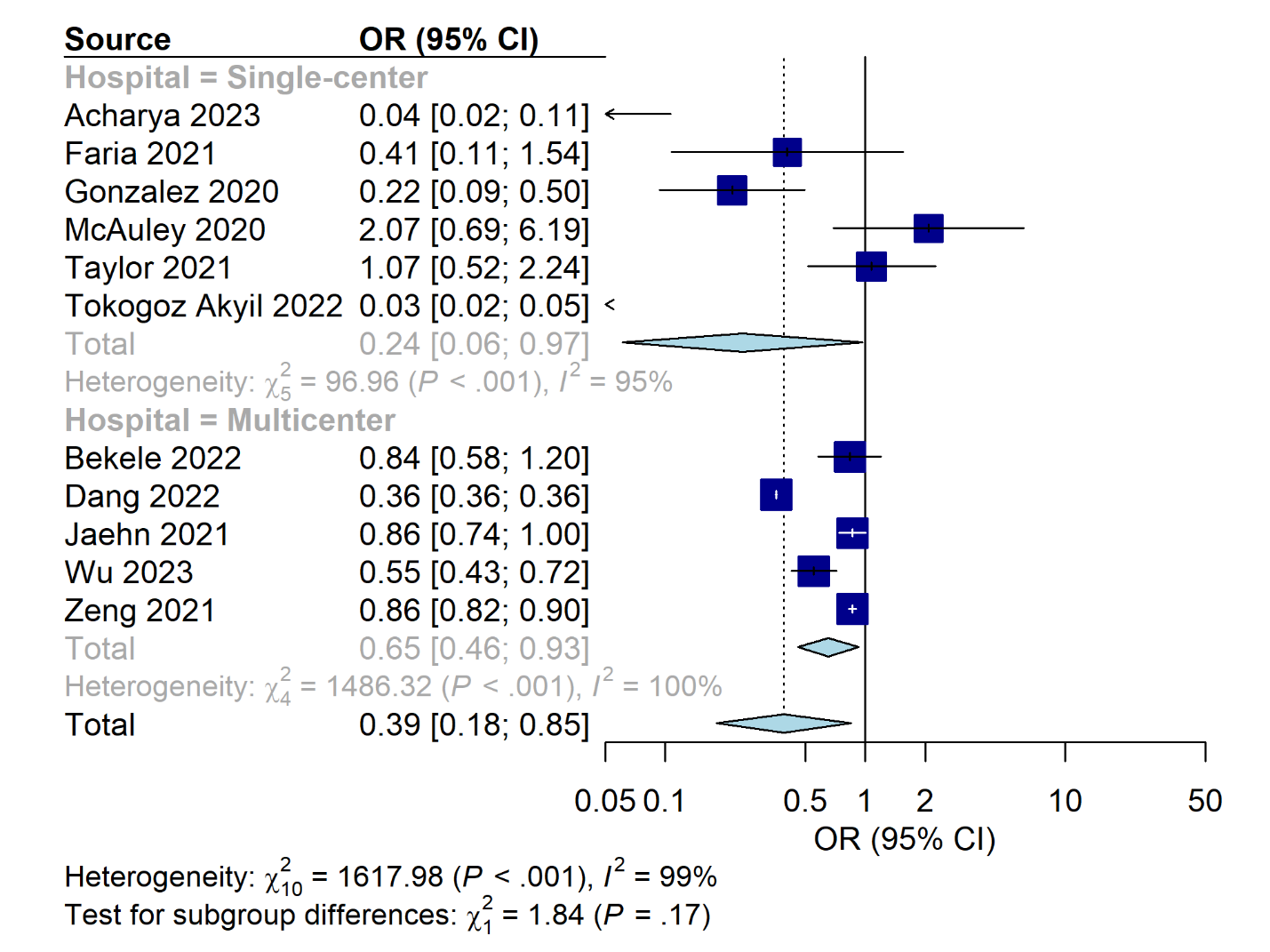


(A) No. of hospitals conducting a study


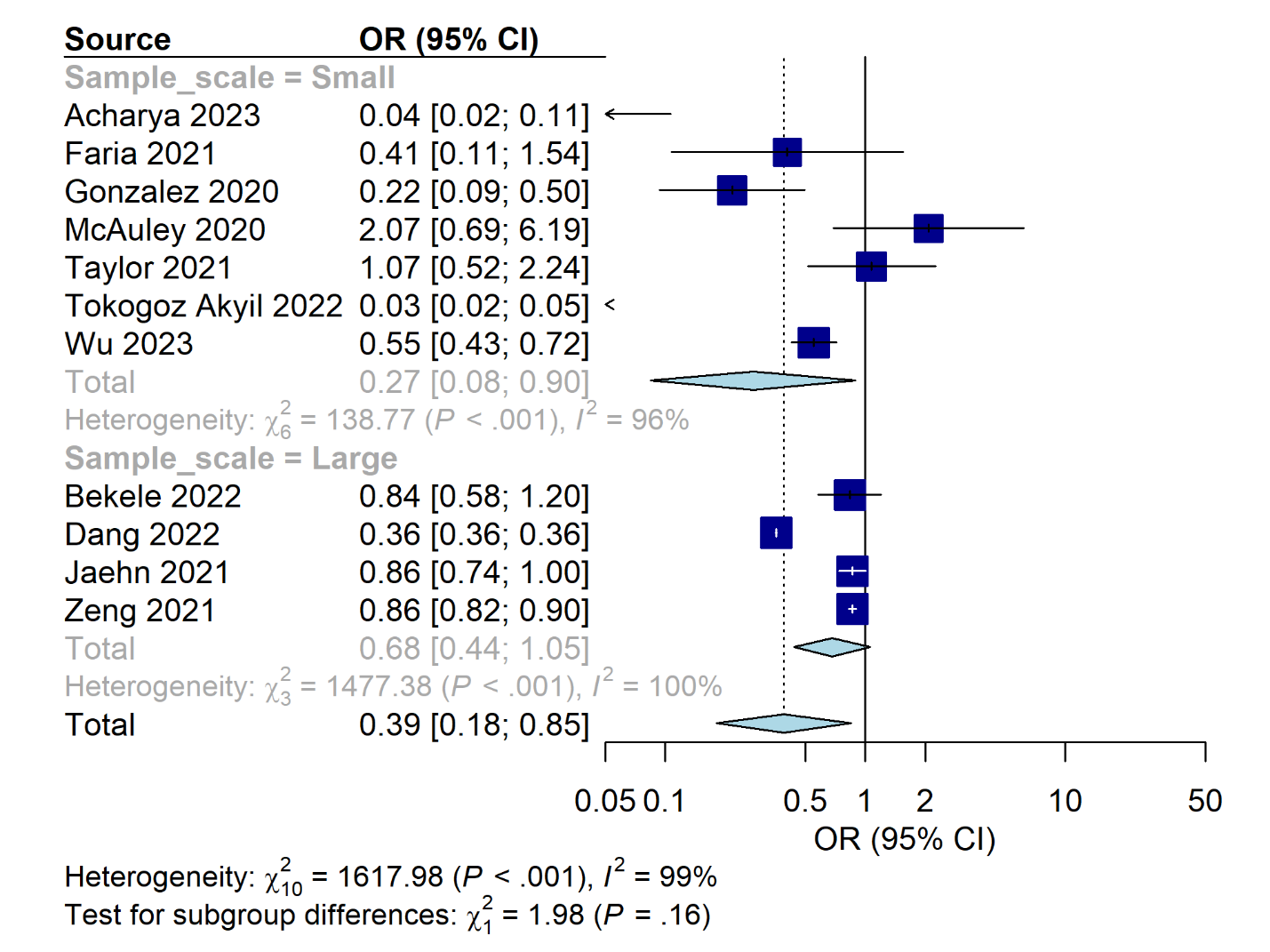


(B) Sample size


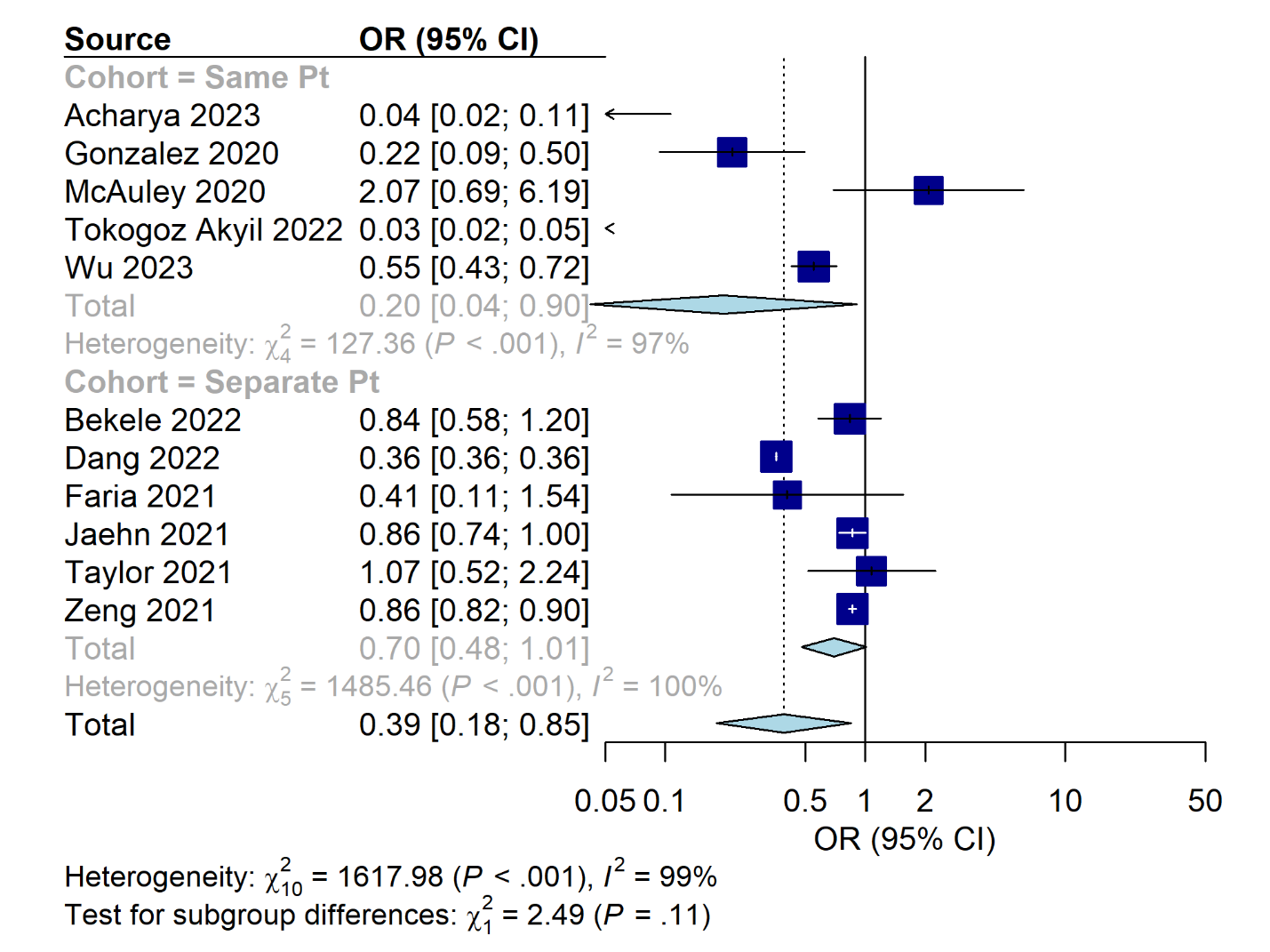


(C) Article type


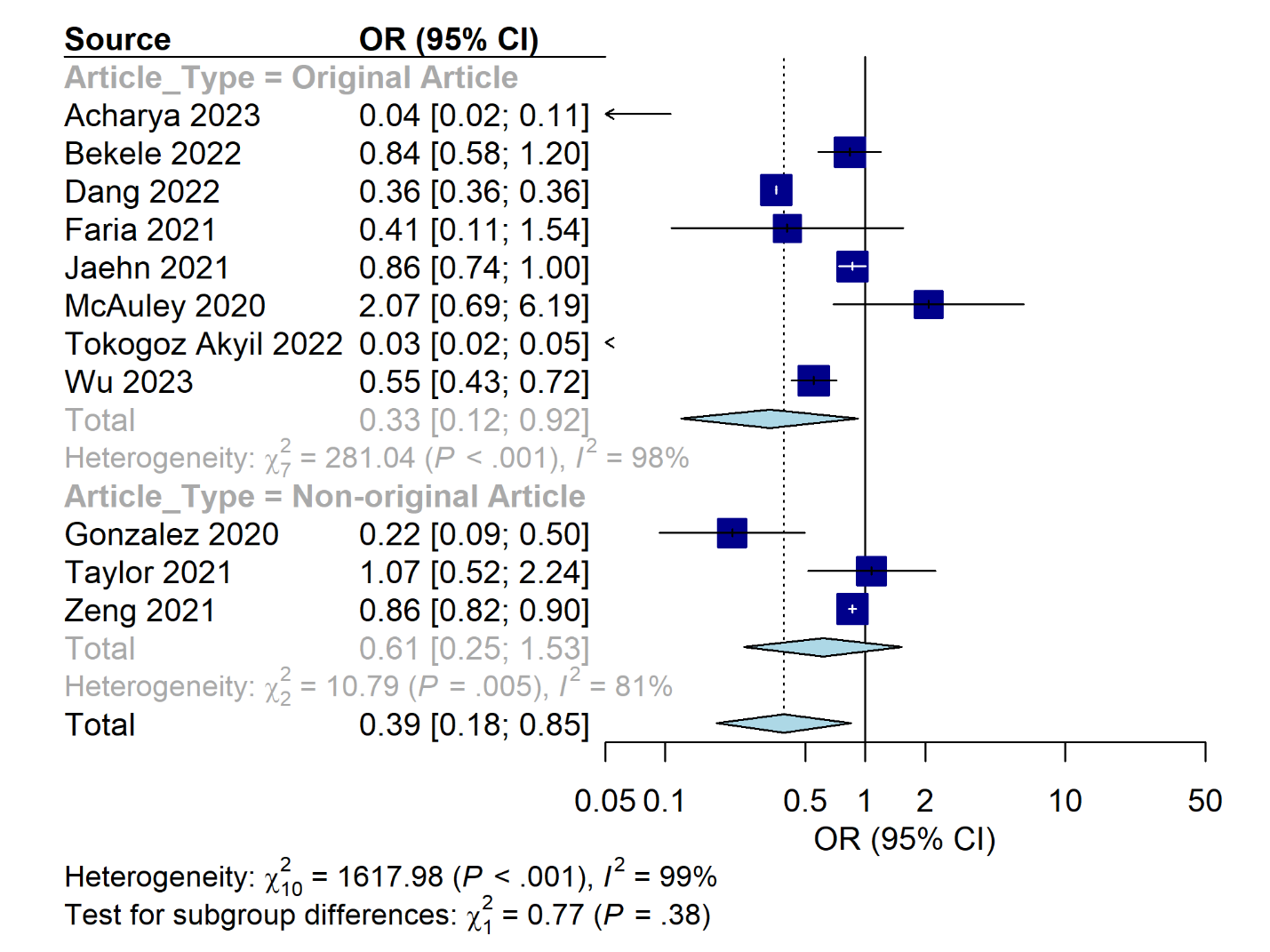


(D) Sameness cohort.

**Supplementary Figure S3.** Funnel plot. (A) Mortality and (B) Hospitalization.


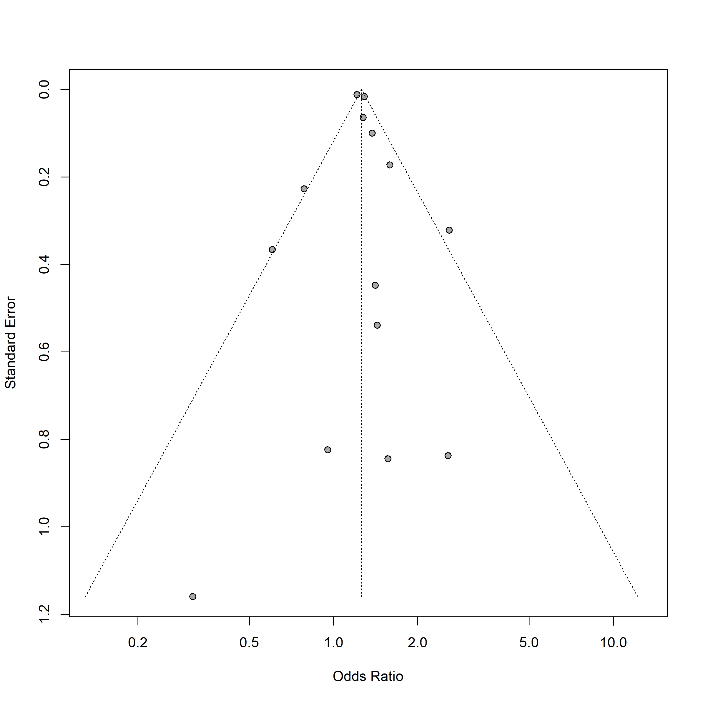


(A) Mortality


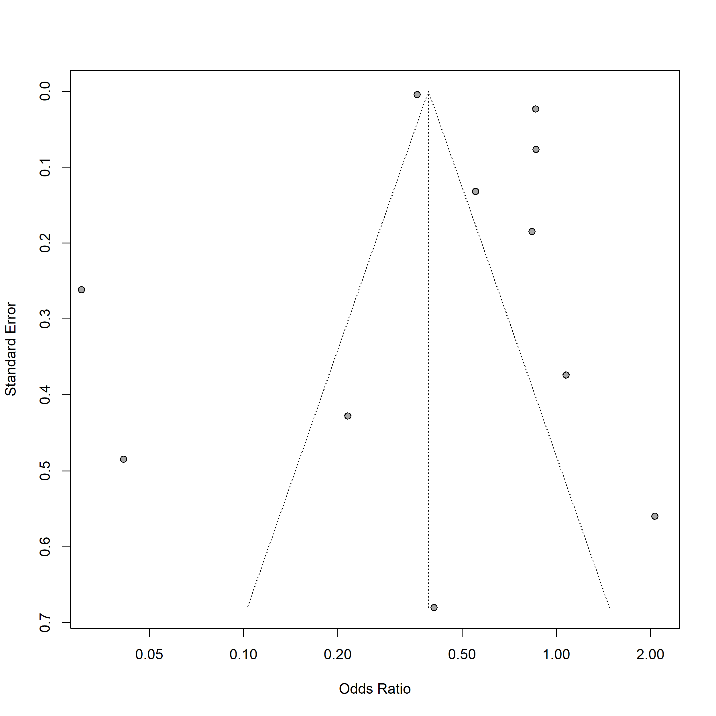


(B) Hospitalization.
